# Mucoadhesive-to-penetrating controllable peptosomes-in-microspheres co-loaded with anti-miR-31 oligonucleotide and Curcumin for targeted colorectal cancer therapy

**DOI:** 10.7150/thno.40318

**Published:** 2020-02-18

**Authors:** Ran Zhao, Sujuan Du, Ying Liu, Cong Lv, Yongli Song, Xinchun Chen, Bing Zhang, Dan Li, Shan Gao, Wei Cui, Maksim V. Plikus, Xiaohua Hou, Kaichun Wu, Zhanju Liu, Zhihua Liu, Yingzi Cong, Yuan Li, Zhengquan Yu

**Affiliations:** 1 State Key Laboratories for Agrobiotechnology and Beijing Advanced Innovation Center for Food Nutrition and Human Health, College of Biological Sciences, China Agricultural University, Beijing, 100193, China.; 2 Guangdong Provincial Key Laboratory of Regional Immunity and School of Medicine, Shenzhen University, Shenzhen, 518055, China.; 3 Beijing Advanced Innovation Center for Food Nutrition and Human Health, Key Laboratory of Functional Dairy, College of Food Science and Nutritional Engineering, China Agricultural University, Beijing, 100083, China.; 4 College of Veterinary Medicine, China Agricultural University, Beijing, 100193, China.; 5 CAS Key Laboratory of Bio-medical Diagnostics, Suzhou Institute of Biomedical Engineering and Technology, Chinese Academy of Sciences, Suzhou, Jiangsu, 215163, China.; 6 Institute of Reproductive and Developmental Biology, Faculty of Medicine, Imperial College London, W12 0NN, UK.; 7 Department of Developmental and Cell Biology, Sue and Bill Gross Stem Cell Research center, Center for Complex Biological Systems, University of California, Irvine, Irvine, CA 92697, USA.; 8 Division of Gastroenterology, Union Hospital, Tongji Medical College, Huazhong University of Technology and Science, Wuhan, 430022, China.; 9 Department of Gastroenterology, Xijing Hospital, The Fourth Military Medical University, Xi'an, 710032, China.; 10 Department of Gastroenterology, The Shanghai Tenth People's Hospital, Tongji University, Shanghai, 200072, China.; 11 State Key Laboratory of Molecular Oncology, National Cancer Center/Cancer Hospital, Chinese Academy of Medical Sciences, Beijing, 100021, China.; 12 Department of Microbiology and Immunology, University of Texas Medical Branch, Galveston, TX 78701, USA.

**Keywords:** Peptosomes-in-microsphere, Mucoadhesive-to-penetrating, Rectal delivery, Oral delivery, Anti-microRNA oligonucleotide, Anti-colorectal cancer therapy.

## Abstract

**Background:** Accumulating evidences indicate that nanomedicines greatly decrease the side effects and enhance the efficacy of colorectal cancer (CRC) treatment. In particular, the use of rectal delivery of nanomedicines, with advantages such as fast therapeutic effects and a diminishing hepatic first-pass effect, is currently emerging.

**Method:** We established a CRC targeted delivery system, in which α-lactalbumin peptosomes (PSs) co-loaded with a microRNA (miR)-31 inhibitor (miR-31i) and curcumin (Cur) were encapsuslated in thiolated TEMPO oxidized Konjac glucomannan (sOKGM) microspheres, referred as sOKGM-PS-miR-31i/Cur. The CRC targeting capability, drug release profiles, mucoadhesive-to-penetrating properties and therapeutic efficacy of sOKGM-PS-miR-31i/Cur delivery system were evaluated in colorectal cancer cells and azoxymethane-dextran sodium (AOM-DSS) induced tumor models.

**Results:** sOKGM-PS-miR-31i/Cur delivery system were stable in the harsh gastrointestinal environment after rectal or oral administration; and were also mucoadhesive due to disulfide bond interactions with the colonic mucus layer, resulting in an enhanced drug retention and local bioavailability in the colon. Concomitantly, the released PS-miR-31i/Cur PSs from the microsphere was mucus-penetrating, efficiently passing through the colonic mucus layer, and allowed Cur and miR-31i specifically target to colon tumor cells with the guide of CD133 targeting peptides. Consequently, rectal delivery of sOKGM-PS-miR-31i/Cur microspheres suppressed tumor growth in an azoxymethane-dextran sodium sulfate (AOM-DSS)-induced tumor model.

**Conclusion:** sOKGM-PS-miR-31i/Cur microspheres are effective rectal delivery system with combined advantages of mucoadhesive and mucus-penetrating properties, representing a potent and viable therapeutic approach for CRC.

## Introduction

Colorectal cancer (CRC) is the leading cause of cancer related death worldwide 1. The pathogenesis of CRC is complicated, which is attributed to the dysregulation of multiple signaling pathways like Wnt, TGFβ and Ras 2-6 , as well as microRNAs 7, making it difficult to develop effective targeted drugs for CRC treatment. Currently, surgical excision combined with chemotherapy is a common therapeutic strategy for CRC 8. However, the efficacy of current treatments is compromised as the drug molecule does not reach the target site with an effective concentration, and the low specificity of chemotherapeutics often produces a range of dose-limiting side effects including hair loss, nausea and vomiting 9, 10. Thus, it is critical to develop effective targeted drugs for CRC with advantages of high efficacy and low side effects.

Accumulating evidences indicate that microRNAs play important roles in regulating tumorigenesis and have potential as therapeutic targets 11-14. Among microRNAs, miR-31 has been characterized as an oncogene in a variety of human cancers including breast, lung and colon cancers. miR-31 exhibits markedly upregulated expression in CRC 15-18 and promotes tumorigenesis by activating Wnt, and concomitantly suppressing BMP and TGFβ signaling pathways, which are critical for CRC initiation and development 19. Given the critical role of miR-31 in CRC, anti-miR-31 oligonucleotide, i.e., miR-31 inhibitor (miR-31i), is identified as a potential high-value therapeutic for CRC. Particularly, small RNA-based nanosystems are increasingly developed in the treatment for a variety of diseases 20. Therefore, it becomes very promising to make a miR-31i based nanosystem for treating CRC. In addition, curcumin (Cur) is a great natural anti-cancer agent, suppressing colorectal cancer cell proliferation and migration by regulating multiple signaling pathways, with advantages of low toxicity and good biocompatibility. Due to its poor aqueous solubility, Cur is usually loaded to nanocarriers for CRC treatment 21-24. Considering distinct mechanisms of miR-31i and Cur in suppressing CRC development, targeted co-delivery of miR-31i and Cur with nanocarriers to colorectal tumor sites might represent an effective approach for CRC treatment via synergistic suppression of tumor growth.

The nanoparticle-based therapeutic strategies have spurred substantial advances in decreasing side effects and enhancing efficacy for treating CRC 25-28. Over the past few years, several types of nanoparticles (NPs) have been developed to deliver chemotherapeutic drugs for CRC, including liposome-based NPs, biodegradable and noncytotoxic polymeric NPs 29-33. However, nonspecific uptake of these nanomedicines still results in adverse effects and toxicity on organs like liver and kidney following distribution of drug in blood circulation around the body, since they are generally designed to achieve systemic delivery of therapeutics via parenteral route. Recently, the use of rectal delivery of nanomedicines for CRC is emerging with advantages such as fast therapeutic effects and diminishing hepatic first-pass effect 34. Despite these obvious advantages, the development of rectal delivery system is still slow due to many critical challenges, as nanosystems typically undergo rapid depletion toward the exterior following administration in the rectum as a result of the natural bowel movement and defecation reflex 35. Two types of rectal delivery nanosystems, mucoadhesive and mucus-penetrating types have been developed. Mucoadhesive nanosystem, which can adhere to colonic mucus, benefit enhancing drug retention and improving local drug bioavailability, but the strong adhesion to mucins impairs effective transport across the inner layer of mucus thus limiting drug absorption 36, 37. By comparison, mucus-penetrating nanosystems can easily pass through the inner layer of mucus, but its drug retention time is short 38. A smart drug delivery system which can switch from mucoadhesive to mucus-penetrating at the time needed is ideal for treating CRC. In addition, another challenge is that those biocompatible biomaterials of nanosystems were unstable under gastrointestinal conditions, while chemical materials are toxic and have stimulatory effects on immune system 39, 40. Formulation into nanoparticles have potential for improving drug stability in the harsh gastrointestinal (GI) tract environment, providing opportunities for targeting specific sites in the GI tract, increasing drug solubility and bioavailability, and providing controlled release properties in the GI tract 41. Therefore, it is critical to make an ideal rectal delivery system for CRC to overcome these challenges.

In order to make an efficient rectal delivery system for CRC, we first developed mucus-penetrating NPs of α-lactalbumin (α-La) PSs targeting a colon cancer stem cell antigen CD133 and co-loaded with miR-31i and Cur. Then the mucus-penetrating NPs were further incorporated into thiolated TEMPO oxidized Konjac glucomannan (sOKGM) microspheres, which possessed ideal mucoadhesive property due to disulfide bond interactions with the colonic mucus layer 42-45, forming sOKGM-PS-miR-31i/Cur composite microspheres. The microspheres appeared long drug retention, stable in gastrointestinal conditions, pH responsive and low toxicity. Concomitantly, the released PS-miR-31i/Cur NPs from the microsphere efficiently passing through the colonic mucus layer, and allowed Cur and miR-31i to target to colon tumor cells, thus suppressing colon tumor growth efficiently. The sOKGM-PS-miR-31i/Cur microspheres may represent as a novel and viable rectally targeted therapeutic approach for CRC with high efficacy and low toxicity.

## Materials and methods

### Materials

α-lactalbumin (≥85%), curcumin (≥98%), cysteine (≥97%), N-(3-dimethylaminopropyl)-N′-ethylcarbodiimide hydrochloride crystalline (EDC), pepsin and trypsin were purchased from Sigma-Aldrich, USA. *Bacillus licheniformis* protease (BLP) was a kindly gift from Novozymes, Denmark. The CD133-targeting peptide (TP, KMPKEVPSSWLS) was synthesized by GL Biochem (Shanghai) Ltd., China. The miR-31i (5'-AGC UAU GCC AGC AUC UUG CCU-3') was synthesized by Suzhou GenePharma, China. Konjac glucomannan (≥98%) polymer was purchased from Yizhimoyu Co., China. Hydrophobic Cy3, hydrophilic Cy3-SE and hydrophilic Cy7-SE were purchased from Fanbo Biochemical Co., China. FeSO_4_·7H_2_O, hexane, methanol, and other chemical reagents were purchased from Beijing Chemical Reagent Factory, China. Ultrapure water was purified by Milli-Q^®^ Reference Water Purification System (Sigma-Aldrich, USA).

### Cell lines and animals

The HCT116, LoVo and HT29-MTX human CRC cell lines were purchased from American Type Culture Collection (ATCC). Cells were cultured at 37°C in DMEM supplemented with 10% FBS. C57BL/6 mice were purchased from Charles River Co., China.

### Formulation of sOKGM-PS delivery system

#### Preparation of PS-TP-miR-31i/Cur NPs

The synthesis of PS-TP-miR-31i/Cur NPs was optimized based on a previously described method 46 with modifications. α-La (1 mg/mL) was dissolved in Tris-HCl buffer at pH 7.5 and hydrolyzed by BLP (at a 1:25 BLP:α-La weight ratio) for 30 min at 50°C to generate α-La peptides. PSs were self-assembled from α-La peptides at 4°C overnight. The anticancer agent Cur (10 mg/mL, dissolved in DMSO) can be loaded into the PS cores by mixing Cur with PS buffer solution (at a 6:1 PS:Cur weight ratio) during the process of self-assembly from α-La peptides. After Cur being loaded into PS core, the Tris-HCl buffer and DMSO was removed from formulation by dialyzed the 6 mL (1 mg/mL, equivalent to the PS concentration) PS-Cur NPs solution with 1 L ultrapure water for 30 minutes, and it was repeated four times. The CD133-TP (KMPKEVPSSWLS) was conjugated to active carboxyl groups on the surface of the PSs after EDC activation at room temperature for 8 hours (at a 5.5:4:1 PS:TP:EDC weight ratio). Excess EDC and TPs were removed using a centrifugal filter tube (10 kDa, Millipore Co., Germany) at 3,500 rpm for 30 min. The miR-31i was mixed with PS-TP-Cur NPs (at a 60:1 PS:miR-31i weight ratio) in DNase/RNase free water to generate PS-TP-miR-31i/Cur NPs. The PS-TP-miR-31i/Cur NPs were directly used in subsequent experiments or freeze dried for storage at -20°C.

#### Preparation of sOKGM-PS-miR-31i/Cur microspheres

TEMPO-OKGM with a degree of oxidation of 80% (DO80) was prepared following our previously described method 47. We first added cysteine to OKGM polymers. In this process, 120 mg of DO80 OKGM was completely dissolved in 20 mL pH 3.0 HCl aqueous solution; then, 9.98 mg of EDC and 9.14 mg of cysteine were dissolved in 200 μL of water and added dropwise into the OKGM solution with mild stirring at 25°C for 24 hours. Excess cysteine and EDC were removed by ethanol dialysis. Cysteine-linked OKGM polymers were freeze dried and stored at -20°C. A previously described inverse emulsion method 47 was used to generate microspheres. Fifty milligrams of cysteine-linked OKGM polymers and 12 mg of FeSO4·7H2O were dissolved in 1 mL of water, and 1.5 g of Span 80 was dissolved in 40 mL of paraffin oil. A 1 mL volume of the aqueous solution and 200 μL of PS-TP-miR-31i/Cur NPs (30 mg/mL, equivalent to the PS concentration) were added dropwise into the oil phase with mild stirring at 35°C for 4 hours. Consequently, cysteine-linked OKGM polymers were crosslinked by both Fe^3+^ ions coordinated with carboxyl groups and disulfide bonds to form the microspheres (sOKGM), and PS-TP-miR-31i/Cur NPs were concomitantly encapsulated in the sOKGM microspheres. Next, the sOKGM-PS-miR-31i/Cur microspheres were washed three times with hexane and then three times with methanol. In each washing step, the system was centrifuged (3000 rpm, 2 min), the liquid was decanted, and the particles were resuspended. In these processes, hexane was removed by washing with methanol. After the last step, the microparticles were washed by pH 3.0 HCl aqueous solution for another three times to remove methanol. Finally, the sOKGM-PS-miR-31i/Cur microspheres were dissolved in 4 mL of pH 3.0 HCl aqueous solution for storage.

### Characterization of sOKGM-PS delivery system

#### Characterization of PS-TP-miR-31i/Cur NPs

The morphological characteristics of PS-TP-miR-31i/Cur NPs were observed using transmission electron microscopy (TEM, JEM-1400, Japan). Samples were diluted with water to a concentration of 0.1 mg/mL (equivalent to the PS concentration), placed on a 200-mesh copper grid, stained with uranyl acetate for 1 min, and then air dried at room temperature. The size and zeta potential of PSs, PS-TPs, PS-TP-miR-31i NPs and PS-TP-miR-31i/Cur NPs were determined by a Nano-ZS 2000 (Malvern Instruments, Ltd., U.K.). For the dispersion stability test, 1 mg/mL PS-TP NP solution (equivalent to the PS concentration) was prepared in water and incubated in a 37°C incubator. Then, the size distribution and zeta potential were measured daily for 7 consecutive days.

#### Loading efficiency of the miR-31i

FAM-labeled miR-31i sequences were mixed with PS-TP NPs at increasing weight ratios (PS:miR-31i weight ratios of 10:1, 20:1, 40:1, and 60:1) to generate PS-TP-miR-31i NPs. Gel shift assays were conducted on 1.5% agarose gels. The results were visualized and imaged using a standard imaging system (Bio-Rad Laboratories, USA).

#### Determination of thiol groups in sOKGM polymers

The substitution degree of thiol group in the sOKGM polymers was determined by Ellman's method according to the manufacturer's instructions. Briefly, 0.4 mg/mL of sOKGM polymers solution was prepared and diluted with 0.1 M sodium phosphate buffer (pH 8) containing 1 mM EDTA to prepare different dilutions. 16 mL aliquots of each dilution were added to 180 mL of 0.5 M phosphate buffer (pH 8.0) and 4 mL of Ellman's reagent (4 mg/mL of DTNB in 0.5 mol/L phosphate buffer, pH 8.0). Control reactions were carried out with non-modified OKGM polymers. The samples were shielded from light and incubated at room temperature for 15 min. Then, 200 μL of the supernatant was transferred to a micro titration plate and the absorbance was measured at a wavelength of 412 nm with an Infinite 200 PRO multimode reader (Tecan, Switzerland). The amount of free thiol groups was calculated from the standard plot prepared by measuring the absorbance of L-cysteine hydrochloride monohydrate solutions as described above.

#### Size distribution of sOKGM-PS-miR-31i/Cur microspheres

The sizes of sOKGM-PS-miR-31i/Cur microspheres were determined by the Nano-ZS 2000 (Malvern Instruments, Ltd., U.K.). For the dispersion stability test, 1.5 mg/mL (equivalent to the PS concentration) sOKGM-PS-miR-31i/Cur was prepared in aqueous solution at pH 3.0 and incubated in a 37°C incubator. The size distribution of thek9 sOKGM-PS-miR-31i/Cur microspheres was measured daily for 7 consecutive days. The measurements were performed in triplicate.

#### Characterization of sOKGM-PS-miR-31i/Cur microspheres

PS-TP NPs were labeled with Cy3 (red) on carboxyl groups, and miR-31i sequences were labeled with FAM (green) in sOKGM-PS-miR-31i/Cur microspheres. The morphology of the sOKGM-PS-miR-31i/Cur microspheres was observed using a fluorescence microscope (Nikon, Japan) and a confocal laser scanning microscope (Nikon, Japan).

### Stability and retention assay

#### *In vitro* stability and controlled release assay

The stability of disulfide bond/Fe^3+^-mediated OKGM (sOKGM)-PS-miR-31i/Cur microspheres was determined by microscopy (Nikon, Japan) after incubation in simulated gastric or intestinal conditions at 37°C. Drug release studies of sOKGM-PS-miR-31i/Cur microspheres were carried out by centrifugation. PS-TP-miR-31i/Cur-encapsulating Fe^3+^-mediated OKGM (fOKGM) or sOKGM microspheres were immersed in simulated gastric solution and simulated intestinal solution or in water at different pH values (from pH 3.0 to pH 8.0), and then placed in a 37°C incubator. At predetermined time intervals, samples were collected then the OKGM microspheres and nanoscaled peptsomes were separated by centrifugation where the released PS-Cur presented in supernatant. The released PS-Cur from microspheres in supernatant can be observed by TEM, and the concentration of Cur extracted from PS-Cur by acetonitrile in the supernatant of each sample was measured by ultraviolet (UV) absorbance at 445 nm using an ultraviolet spectrophotometer (RF-6000, Shimadzu, Japan). The simulated gastric solution contained 3.2 g/L pepsin and 2.0 g/L NaCl. The final pH value was adjusted accordingly to 1.5 with HCl prior to the addition of pepsin. The simulated intestinal solution contained 10 g/L trypsin, 5.59 g/L K_2_HPO_4_, and 0.41 g/L KH_2_PO_4_. The final pH value was adjusted accordingly to 7.5 with NaOH prior to the addition of trypsin.

#### *In vivo* retention and distribution assay

PSs were labeled with Cy7-SE as a fluorescent tracker. A total of 100 μL of 1.5 mg/mL (equivalent to the PS concentration) PS-Cy7 NPs, fOKGM-PS-Cy7 and sOKGM-PS-Cy7 microspheres were rectal or gavage administered to 6-week-old C57BL/6 male mice, which were pre-fasted for 24 hours. At a predetermined time after administration, the mice were sacrificed for sample collection. The Cy7 fluorescence signals in the gastrointestinal (GI) tract were examined using an IVIS Spectrum (Spectral Instruments Inc., USA).

### Toxicity analysis

#### *In vivo* toxicity analysis

6-week-old C57BL/6 male mice were gavage-treated with 100 μL of PBS or sOKGM-PS (1.25 mg/mL, equivalent to the PS concentration) once daily for 7 consecutive days. After the treatment, the mice were sacrificed for sample collection. Colon, intestine, liver and kidney samples were collected, fixed in 10% formalin, and paraffin embedded. Five-micrometer sections were stained with hematoxylin and eosin (H&E). For immunohistochemistry, sections were deparaffinized, pretreated with 0.01 M citrate buffer (pH 6.0), and incubated first with primary antibodies and then with secondary antibodies. The signals were detected by diaminobenzidine (DAB). Hematoxylin was used as a counterstain. Anti-p65 and p-STAT3 antibodies (1:1000, Cell Signaling Technology, USA) were used to detect inflammatory signaling pathways.

### *In vitro* cytotoxicity analysis

HCT116 and LoVo cells were cultured in 96-well plates (Corning Inc., USA) at a density of 5,000 cells per well for 24 hours. Then, the cells were incubated in fresh DMEM containing PS, TP, PS-TP, miR-31i, Cur, PS-miR-31i, PS-Cur, PS-miR-31i/Cur, PS-TP-miR-31i or PS-TP-miR-31i/Cur at concentrations of 24, 48, 72, and 96 μg/mL (equivalent to the PS concentration) for an additional 24 hours. Cell viability was measured using a Cell Counting Kit-8 (CCK-8, Beyotime Biotechnology, China). A 10 μL volume of CCK-8 solution was added to each well, and the cells were incubated at 37°C for an additional 1 hour. The absorbance values of each well at 450 nm, which are proportional to the number of viable cells in the well, were read on an ELx800 microplate reader (BioTek Inc., USA).

### The analysis on mucoadhesive and mucus-penetrating features of sOKGM-PS delivery system

#### *In vivo* analysis on adhesion and penetration of sOKGM-PS delivery system to colon mucins

Cy3 was loaded into PSs as a fluorescent tracker. A total of 100 μL of 1.5 mg/mL (equivalent to the PS concentration) sOKGM-PS-Cy3 or fOKGM-PS-Cy3 microspheres and 0.1 μL of wheat germ agglutinin (WGA,1 mg/mL, Sigma-Aldrich, USA) were administered to 8-week-old C57BL/6 male mice by enema. Whole-mount colon tissue was directly attached to slides and observed by a confocal laser scanning microscope (Nikon, Japan) after biopsy. Colon confocal laser scanning microscopy (CLSM) tomography was performed at 1 μm per layer.

#### *In vitro* analysis on adhesion of sOKGM microspheres to HT29-MTX cells

The mucus-secreting cell line HT29-MTX was cultured in 6-well plates (Corning Inc., USA). sOKGM-PS-Cy3 or fOKGM-PS-Cy3 microspheres were dissolved in DMEM supplemented with 10% FBS and were incubated with 90% confluent HT29-MTX cells for 30 min at 37°C. After incubation, the cells were washed twice with PBS to remove unbound microspheres. Then, the cells were observed by CLSM (Nikon, Japan). The Cy3 signal coverage in the mucus surface of HT29-MTX cells was measured using ImageJ 48, 49.

#### *In vivo* analysis on penetration of PS NPs to colon mucins

PS NPs and sOKGM microspheres were labeled by Cy3. A total of 100 μL of 1.5 mg/mL (equivalent to the PS concentration) PS-Cy3 NPs or fOKGM-Cy3 microspheres and 0.1 μL of wheat germ agglutinin (WGA,1 mg/mL, Sigma-Aldrich, USA) were administered to 8-week-old C57BL/6 male mice by enema. Whole-mount colon tissue was directly attached to slides and observed by a confocal laser scanning microscope (Nikon, Japan) after biopsy at predetermined time points. Colon confocal laser scanning microscopy (CLSM) tomography was performed at 1 μm per layer.

### Cellular uptake assay

Liposoluble Cy3 (representing Cur) was loaded into the core of PS-TP-miR-31i NPs, and miR-31i was labeled with FAM (green). HCT116 or LoVo cells were incubated with 48 μg/mL (equivalent to the PS concentration) PS-TP-miR-31i/Cy3 or PS-miR-31i/Cy3. The cells were collected at predetermined time points, suspended in 0.3 mL of PBS, and analyzed by flow cytometry. After 12 hours of incubation, the cells were used for CLSM imaging (Nikon, Japan).

### Quantitative RT-PCR (qRT-PCR)

Total RNA was isolated from HCT116 or LoVo cells using TRIzol reagent (Life Technologies, USA) after incubation with 48 μg/mL (equivalent to the PS concentration) PS-TP-miR-31i. Each RNA sample was reverse transcribed with M-MLV Reverse Transcriptase (Sigma, USA) using oligo (dT) primers. qRT-PCR analysis was performed using Light Cycler 480 SYBR Green I master mix on a Light Cycler 480 real-time PCR system (Roche). The qRT-PCR primers are listed below. U6-forward: 5'-CTC GCT TCG GCA GCA CA-3'; U6-reverse: 5'-AAC GCT TCA CGA ATT TGC GT-3'; miR-31-forward: 5'-ACA CTC CAG CTG GGA GGC AAG ATG CTG GCA-3'; miR-31-reverse: 5'-CTC AAC TGG TGT CGT GGA GTC-3'; Axin1-forward: 5'-TTC TGG GTT GAG GAA GCA GC-3'; Axin1-reverse: 5'-GAT TAG GGG CTG GAT TGG GT-3'; Gsk3β-forward: 5'-CCA ACA AGG GAG CAA ATT AGA GA-3'; Gsk3β-reverse: 5'-GGT CCC GCA ATT CAT CGA AA-3'; Dkk1-forward: 5'-TCC GAG GAG AAA TTG AGG AA-3'; Dkk1-reverse: 5'-CCT GAG GCA CAG TCT GAT GA-3'; Smad3-forward: 5'-ACA GGC GGC AGT AGA TAA CG-3'; Smad3-reverse: 5'-AAC GTG AAC ACC AAG TGC AT-3'; Bmpr1a-forward: 5'- GCT GTC ATC ATC TGT TGT CCT GG-3'; Bmpr1a-reverse: 5'-CAT TAC CAC AAG GGC TAC ACC ACC-3'; Smad4-forward: 5'-GGC TGT CCT TCA AAG TCG TG-3'; Smad4-reverse: 5'-GGT TGT CTC ACC TGG AAT TGA-3'; Tgfbr2-forward: 5'-TTG TTG AGA CAT CAA AGC GG-3'; and Tgfbr2-reverse: 5'-ATA AAA TCG ACA TGC CGT CC-3'.

### Cell proliferation assay

For the cell proliferation assay, HCT116 or LoVo cells were plated in 96-well plates at a density of 100 cells per well in DMEM containing 48 μg/mL (equivalent to the PS concentration) PS-TP, PS-TP-Cur, PS-TP-miR-31i or PS-TP-miR-31i/Cur. Cell proliferation was measured using a CCK-8 assay at serial time points of 0.5, 1, 2 and 3 days.

### Half maximal inhibitory concentration (IC50) assay

HCT 116 cells were incubated with PS-TP-Cur, PS-TP-miR-31i, PS-TP-miR-31i/Cur using 0, 4, 8, 12, 24, 48, 72, 96, 120, 150 and 500 μg/mL (equivalent to PS concentration) doses for 24 hours, and the relative amount of viable cells were estimated by measuring the absorbance of the cell suspension after incubation with MTT assay was carried out and IC50 values of PS-TP-Cur, PS-TP-miR-31i, PS-TP-miR-31i/Cur were analyzed by GraphPad Prism 7.0 software.

### Cell cycle analysis

HCT116 cells were cultured in 6-well plates in DMEM for 24 hours, and the medium was then replaced with fresh medium containing 48 μg/mL (equivalent to the PS concentration) PS-TP, PS-TP-miR-31i, PS-TP-Cur or PS-TP-miR-31i/Cur for an additional 24 hours. Then, the cells were stained with PI/RNase solution (Roche, Switzerland). The cell cycle phases were assessed via flow cytometry.

### Colon tumor mouse model

To generate AOM-DSS-induced colon tumors in mice, 6-week-old C57BL/6 male mice were intraperitoneally injected with AOM (Sigma-Aldrich, USA) at a concentration of 10 mg/kg body weight. After 5 days of AOM injection, the mice were fed drinking water supplemented with 2.5% DSS (molecular weight 36,000-50,000, MP Biomedicals, USA) for 5 days, followed by regular water for a predetermined number of days. After three cycles of DSS treatment, the mice were fed regular water for 28 days. The mice were randomly divided into six groups, of 6 mice per group. Twenty-eight days after the third round of DSS treatment, the mice were enema- or gavage-treated with 100 μL of PBS, miR-31i/Cur, PS-TP-miR-31i/Cur, sOKGM-PS-miR-31i, sOKGM-PS-Cur or sOKGM-PS-miR-31i/Cur (1.25 mg/mL, equivalent to the PS concentration) once daily for 14 consecutive days. After the treatment, the mice were sacrificed for sample collection. The tumor numbers and volumes were evaluated as described above.

### Histology and immunohistochemistry analysis on colon tumors

Colon samples were collected, fixed in 10% formalin, and paraffin embedded. Five-micrometer sections were stained with H&E. For immunohistochemistry, an anti-Ki67 antibody (1:400, Thermo Fisher Scientific, USA) was used to detect cell proliferation. Ki67-positive tumor cells were statistically quantified using microscopy images.

### Statistical analysis

All data were analyzed using GraphPad Prism software (GraphPad, San Diego, CA). All analyses were performed in triplicate or greater and the means obtained were used for independent t-tests. Asterisks denote statistical significance (*p < 0.05; **p < 0.01; ***p < 0.001). All data are reported as mean ± SD. Means and standard deviations from at least three independent experiments.

## Results and discussion

### Preparation and characterization of sOKGM-PS-miR-31i/Cur microspheres

**Figure 1:**
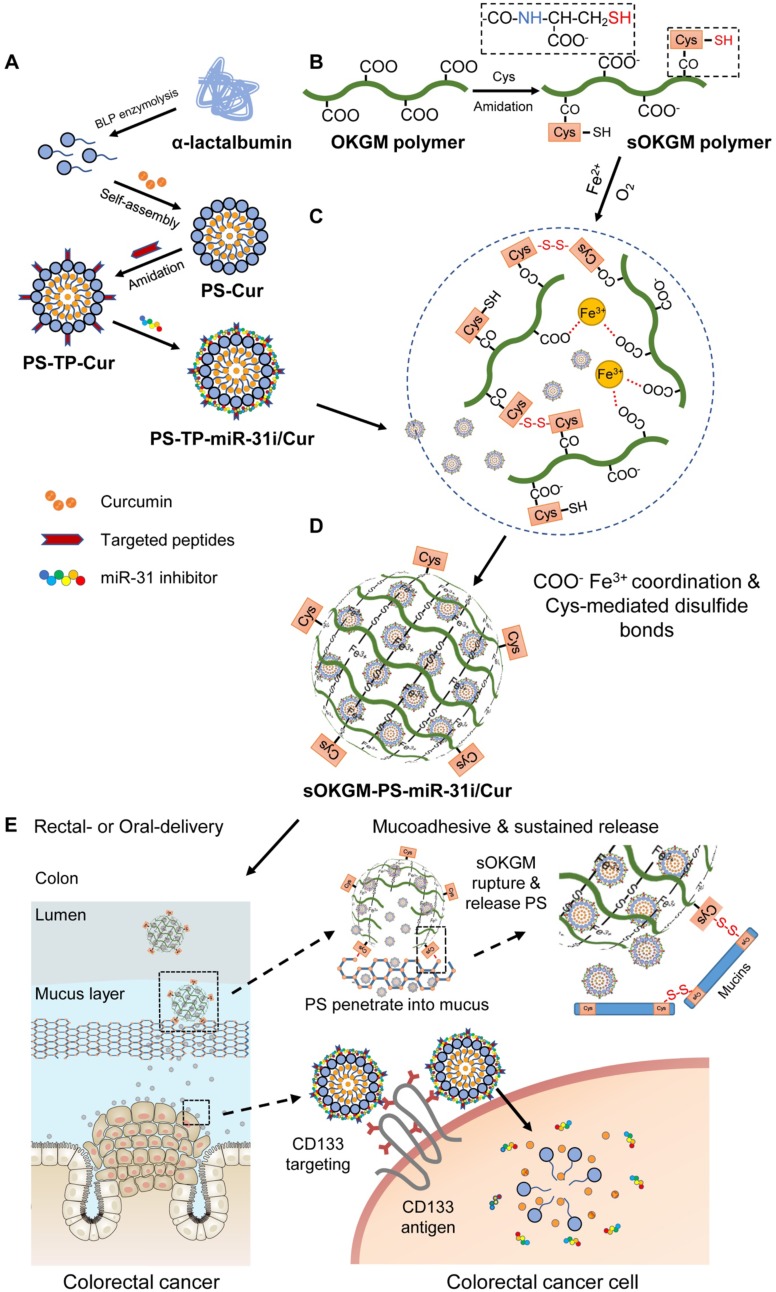
The design and synthesis strategy of cysteines oxidized Konjac glucomannan (sOKGM) microsphere encapsulating CD133 targeting peptide (TP)-linked, miR-31i- and Cur-loaded peptosomes (PSs). (A) Generation of α-Lactalbumin PSs consisting of Cur in the hydrophobic core, and CD133 TP and miR-31i in the surface. (B) Cysteine was linked to carboxyl groups of OKGM polymers via amidation. (C) sOKGM polymers were cross-linked into microsphere via COO^-^ Fe^3+^ coordination and disulfide bonds, forming sOKGM microspheres. (D) TP-linked, miR-31i- and Cur-loaded PSs were encapsulated into sOKGM microspheres, referred as sOKGM-PS-miR-31i/Cur microspheres. (E) sOKGM-PS-miR-31i/Cur microspheres were adhered to colon mucus layer via disulfide bonds providing a sustained release, and the microspheres ruptured due to the instability of Fe^3+^ at neutral pH and then slowly release PS-miR-31i/Cur into the colon cancer cells. These PSs further largely enter the cells through CD133 mediated endocytosis.

The design of peptosomes-in-microspheres delivery system with mucoadhesive-to-penetrating controllable property for treating CRC was presented in Figure 1. Firstly, we generated a CD133 targeted (CD133 targeting peptides (TP)-conjugated) α-lactalbumin (α-La) peptosomes (PSs) co-loaded two anti-tumor reagents - Curcumin (Cur) and anti-miR31 oligonucleotides (miR-31i), which have synergistic effects of suppressing tumor growth, named as PS-TP-miR-31i/Cur (Figure 1A). TP was used to recognize colon cancer stem cell surface antigen CD133 and to enhance the PS NPs uptake by colon cancer cells (Figure 1A). The PS-TP-miR-31i/Cur NPs were further entrapped into cysteines conjugated oxidized Konjac glucomannan (sOKGM) microspheres, which double cross-linked via Fe^3+^ carboxyl coordination and cysteines-mediated disulfide bonds, to form sOKGM-PS-miR-31i/Cur (Figure 1B-D). Then sOKGM-PS-miR-31i/Cur microspheres were delivered into GI tract via rectal or oral routes, and they are relatively stable in gastric conditions while being slowly ruptured at intestinal conditions due to instability of Fe^3+^ at neutral pH. Importantly, due to many cysteines and carboxyl groups on sOKGM polymers, the microspheres preferably adhere to colonic mucus layer. Then the PS-TP-miR-31i/Cur NPs slowly released from the microsphere at colonic tumor sites, penetrated through the mucus layer, and largely internalized by CD133 mediated endocytosis, allowing the release of miR-31i and Cur at the cytoplasm of colorectal cancer cells (Figure 1E). Consequently, the sOKGM-PS-miR-31i/Cur microspheres significantly suppressed colon tumor growth.

**Figure 2:**
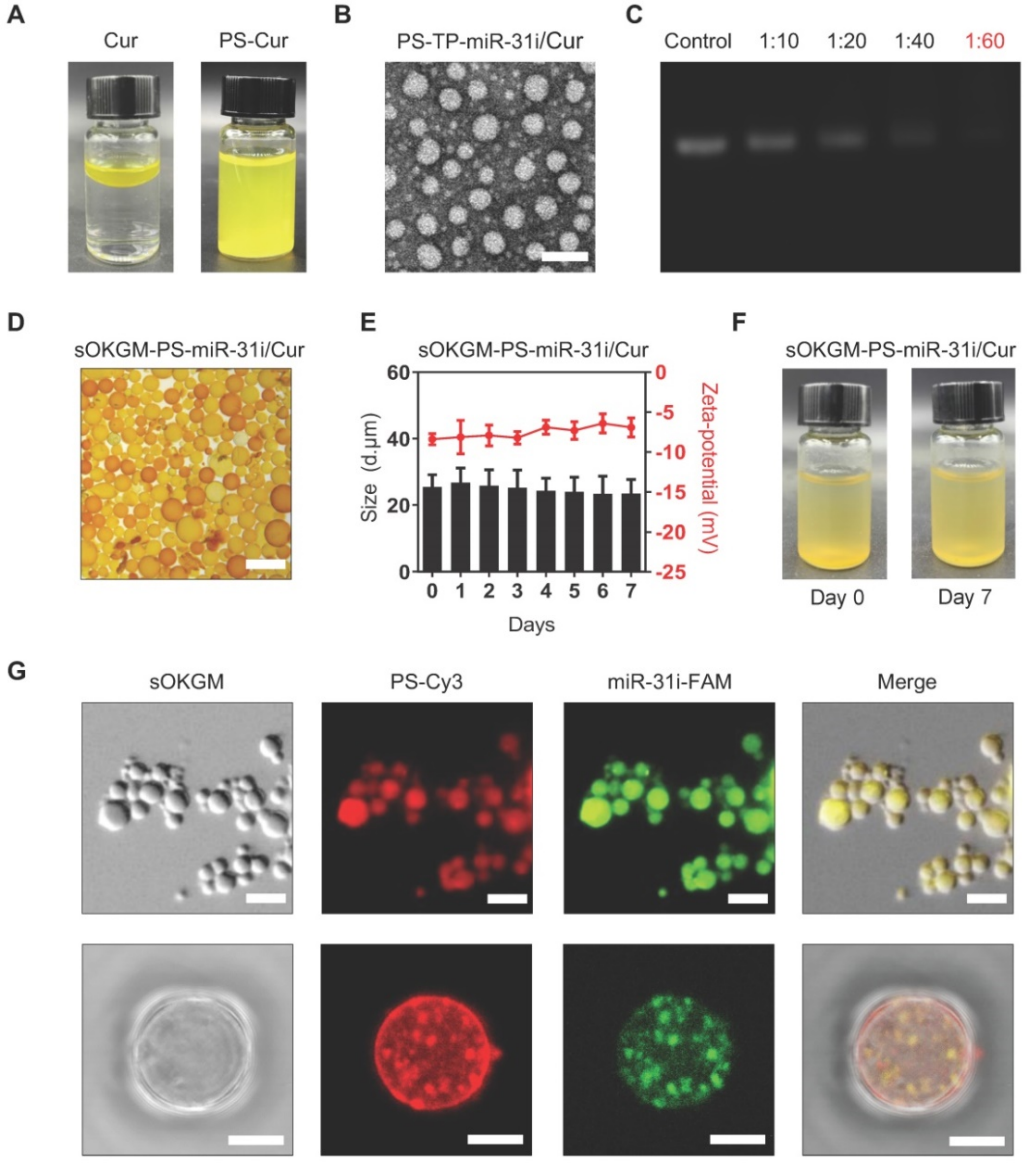
Preparation and characterization of sOKGM-PS-miR-31i/Cur microspheres. (A) Images of the PS-miR-31i/Cur NPs (right) aqueous solution comparing to Cur in water solution (left). (B) TEM images of PS-TP-miR-31i/Cur NPs. Scale bars, 100 nm. (C) Efficiency of miR-31i loading to PS-TP-miR-31i/Cur NPs examined by agarose gel electrophoresis. Panel 1, miR-31i RNA (100 µg/mL); Panel 2 to 5, 1 hour after 100 µg/mL of miR-31i RNA incubating with PS-TP-Cur at concentrations of 1, 2, 4 and 6 mg/mL. The ratio represents miR-31i:PS-TP by weight. Bands: miR-31 inhibitor. (D) Images of sOKGM-PS-miR-31i/Cur microspheres. Scale bars, 50 µm. (E) The size distributions (left) and zeta potential (right) of sOKGM-PS-miR-31i/Cur microspheres over days. The results are reported as the mean ± standard deviation, n = 3. (F) Images of sOKGM-PS-miR-31i/Cur microspheres aqueous solution after 0 and 7 days of storage at 37°C. (G) Microscopy (upper panels) and high magnification confocal (bottom panels) images of sOKGM microspheres encapsulating PSs (labeled by Cy3, red) and miR-31i (labeled by FAM, green). Scale bar, 50 µm (upper panels) and 10 µm (bottom panels).

α-La PSs were formed via the spontaneous-assembly of amphiphilic peptides obtained from partial hydrolysis of α-La by Bacillus licheniformis protease. During this self-assembly process, dimethyl sulfoxide (DMSO)-dissolved Cur was encapsulated into the hydrophobic core of the PSs to form Cur-loaded PSs (PS-Cur) with diameters of approximately 38 nm (Figure 2A and Figure S1A-B) and zeta potential of -14.82 mV (Table S1). Next, CD133 targeting peptides (TP), KMPKEVPSSWLS, were conjugated to the PSs via amidation reaction, forming approximately 53 nm NPs (PS-TP-Cur) (Figure S1C-D). Since the peptides specifically targeted to colon cancer stem cell surface antigen CD133 50, the PSs were guided to colon tumor cells and exhibited high binding affinity to the cells. The size of the PS-TP-Cur NPs appeared stable in water over time (Figure S1E). In addition, the zeta potential of PS-TP-Cur NPs was reversed to approximately +11.49 mV (Table S1) and remained stable over time (Figure S1F) but slightly decreased with an increasing pH from 3 to 8 (Figure S1G). To further enhance the antitumor ability of the NPs, we sought to load miR-31i onto the PS-TP-Cur NP surface. The negatively charged miR-31i were loaded to the surface of PS-TP-Cur by electrostatic interactions, forming PS-TP-miR-31i/Cur NPs (Figure 2B) with the diameter of 68 nm (Figure S1H) and zeta potential of 1 mV (Table S1). The loading efficiency of miR-31i was measured by fluorescence intensity assay. miR-31i bands were not detectable when the weight ratio of PS-TP-Cur (equivalent to PS weight) : miR-31i weight ratio was increased to 60:1, suggesting a high loading efficiency for miR-31i (Figure 2C). The PS-TP-miR-31i/Cur NPs possessed good colloidal stability at 37 ºC during 7-day period (Figure S1I), indicating that the PS-TP can reduce the undesired drug release in the body before reaching to tumor tissues. Collectively, the results showed that we have successfully generated cancer stem cell-targeted PSs to co-deliver miR-31i and Cur.

However, the primary components of PS-TP-miR-31i/Cur NPs, including the α-La peptides, targeting peptides and miR-31i, are easily degraded by proteinase and RNase in GI tract, making this formulation unsuitable for rectal targeted delivery. To solve this problem, we sought to utilize OKGM, which is a nontoxic foodborne polysaccharide 47, to synthesize microsphere as capsules for PS-TP-miR-31i/Cur NPs. To improve its stability in GI tract environment, sulfhydryl groups were added to the OKGM polymers, referred to as sOKGM polymers (Figure 1B). The substitution degree of thiol group in the sOKGM polymers was 52.8 ± 1.7 μmol/g. The sOKGM polymers were then double cross-linked into spheres via both disulfide bonds and COO^-^ Fe^3+^ coordination (Figure 1C and S1J). Furthermore, PS-TP-miR-31i/Cur NPs were successfully encapsulated into sOKGM microspheres (sOKGM-PS-miR-31i/Cur) (Figure 2D). The size and zeta potential of sOKGM-PS-miR-31i/Cur microspheres were stable over time (Figure 2E). The microspheres showed good aqueous solubility for at least 7 days at 37 ºC (Figure 2F). The fluorescent microscopy showed that PSs were encapsulated into sOKGM microspheres (Figure 2G). Collectively, our results demonstrate that the anti-colonic cancer peptosomes-in-microspheres were successfully generated.

### Stability and controlled release of sOKGM-PS-miR-31i/Cur microspheres

**Figure 3:**
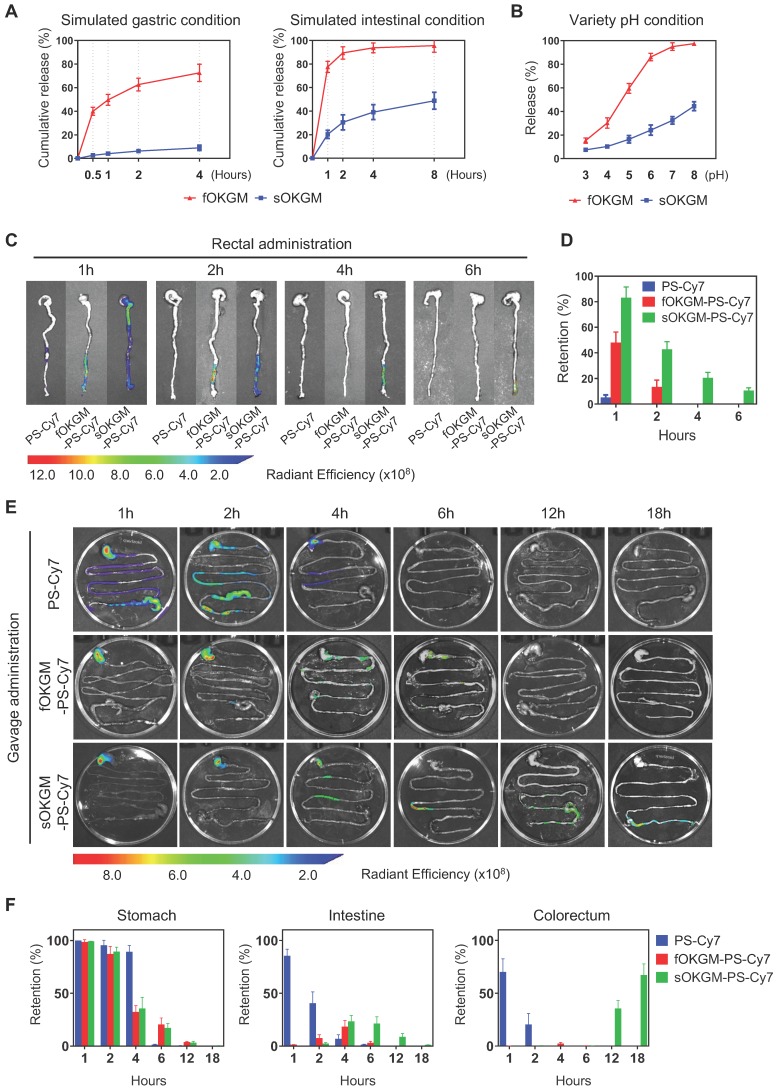
The controlled release and retention of sOKGM-PS microspheres in gastric and intestinal conditions. (A) Percentage of Cur release from sOKGM-PS-miR-31i/Cur and fOKGM-PS-miR-31i/Cur microspheres in simulated gastric and intestinal conditions over time. The results are reported as the mean ± standard deviation, n = 3. (B) Percentage of Cur release from sOKGM-PS-miR-31i/Cur and fOKGM-PS-miR-31i/Cur microspheres in water solutions with pH value at 4 hours. The results are reported as the mean ± standard deviation, n = 3. (C) The distribution of PS-Cy7 NPs, fOKGM-PS-Cy7 and sOKGM-PS-Cy7 microspheres after rectal administration over time, PS labeled by Cy-7. n = 3. (D) Quantification of the Cy7 signal coverage in stomach, intestine and colorectum in panel C. The results are reported as the mean ± standard deviation. (E) The distribution of PS-Cy7 NPs, fOKGM-PS-Cy7 and sOKGM-PS-Cy7 microspheres in gastrointestinal tract after gavage administration over time. PS labeled by Cy-7. n = 3. (F) Quantification of the Cy7 signal coverage in stomach, intestine and colorectum in panel E. The results are reported as the mean ± standard deviation, n = 3.

As a GI tract delivery system, microspheres must be relatively stable under gastric and intestinal conditions. The Fe^3+^ only cross-linked OKGM (fOKGM) microspheres, designed in our previously published work 51, were mostly degraded within 2 hours in simulated gastric condition and completely disappeared within half an hour under simulated intestinal condition (Figure S2A). To improve their stability, disulfide bonds were introduced as the second cross-links by oxidation reaction between cysteine modified OKGM polymers, termed as sOKGM microsphere. The stability of the sOKGM-PS-miR-31i/Cur microspheres was greatly improved and maintained over a period of 4 hours in simulated gastric and intestinal conditions (Figure S2A). Cur was much more slowly released from sOKGM-PS-miR-31i/Cur microspheres than that from fOKGM-PS-miR-31i/Cur microspheres (Figure 3A). TEM images showed that the PSs can be released from sOKGM-PS-miR-31i/Cur or fOKGM-PS-miR-31i/Cur microspheres in simulated gastric and intestinal conditions (Figure S2B). With increasing pH, the release of Cur from sOKGM-PS-miR-31i/Cur microspheres increased gradually, and the maximal release rate is approximately 40% at pH 8.0. In contrast, the release of Cur increased sharply in fOKGM-PS-miR-31i/Cur microspheres, and the release rate approached 100% at pH 7.0, which is similar to the intestinal pH environment (Figure 3B). These findings indicate that sOKGM-PS-miR-31i/Cur microspheres are relatively stable under gastric and intestinal conditions, exhibit drug slowly releasing feature, and possess an intestinal pH-responsive property.

### Distribution and retention of sOKGM-PS-miR-31i/Cur microspheres after rectal and oral administration

Next, we sought to examine the distribution and retention of sOKGM-PS-miR-31i/Cur microspheres in the GI tract of mice after rectal delivery. 1 hour after rectal administration, sOKGM-PS-Cy7 microspheres treatment exhibited the strongest fluorescence signals in the colorectal sites in comparison to fOKGM-PS-Cy7 and PS-Cy7 NPs (Figure 3C-D). The fluorescence signals can last 6 hours in the rectum in the sOKGM-PS-Cy7 microspheres-treated group, while the fOKGM-PS-Cy7 microspheres signal almost disappeared after 2 hours, and individual PS-Cy7 NPs signal disappeared only 1 hour after the treatment (Figure 3C-D). The data showed that sOKGM-PS microspheres can retain the longest in the colorectal sites compared to fOKGM-PS microspheres and barely peptosomes.

Oral administration is a more convenient and acceptable drug delivery route 41, 52; we thus sought to test the potential of sOKGM-PS-Cy7 microspheres being an oral delivery system. To treat CRC, oral-delivered drugs need to pass through the entire GI tract, eventually reaching the colorectal site of tumor localization. Thus, the stability of the delivery system in the GI tract is very important. We found that sOKGM-PS-Cy7 microspheres were stable in the stomach 2 hours after gavage, passed through the intestinal tract within 4 to 12 hours, reached the colon after 12 hours, and maintained in the colorectum until at least 18 hours (Figure 3E-F). In comparison, PS-Cy7 NPs passed through the whole GI tract within 2 hours after gavage, and disappeared after 4 hours of gavage; fOKGM-PS-Cy7 microspheres mostly degraded in the small intestine within 12 hours, and failed to reach to the colon site (Figure 3E-F). 24 hours after sOKGM-PS-Cy3 microsphere gavage, Cy3-labeled PS was released from the microspheres and efficiently targeted colonic cells (Figure S3A), while the Cy3-labeled PS from fOKGM-PS-Cy3 retained in the small intestine (Figure S3B). In summary, our data suggest that sOKGM microspheres can efficiently protect the PS NPs from degradation in the varying GI conditions and render the drug released at the colorectal sites.

After oral treatment of sOKGM-PS-Cy7 microspheres, fluorescence signals were almost not detectable in other organs within 2 hours, while PS-Cy7 strongly distributed to heart, liver, spleen, lung and kidney shortly after intravenous route (Figure S3C). These results indicated that the oral route of sOKGM-PS-Cy7 microspheres makes them have better local drug bioavailability at colorectal sites, reducing their accumulation and potential toxicity to other organs as comparison to intravenous route. In agreement, sOKGM-PS microspheres exhibited low toxicity (Figure S3D) and had no stimulatory effect on immune system (Figure S3E) after oral administration. In summary, our data suggest that sOKGM microspheres have high potential as an oral delivery system of nanomedicines for CRC.

### Mucoadhesive and mucus-penetrating features of sOKGM-PS delivery system

**Figure 4:**
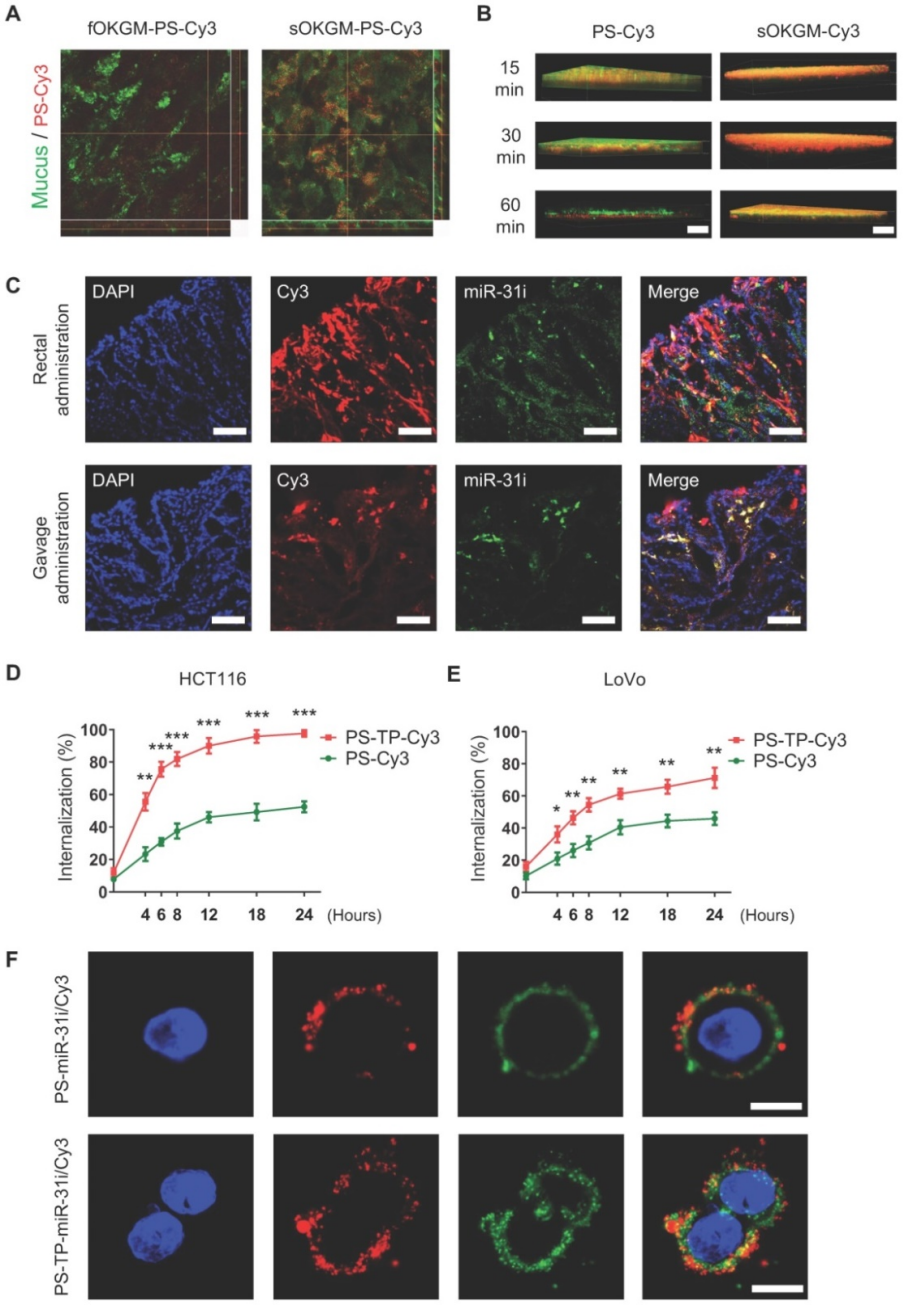
The mucoadhesive-to-penetrating feature and colon cancer cells target efficiency of sOKGM-PS delivery system. (A) CLSM images of fOKGM-PS-Cy3 and sOKGM-PS-Cy3 microspheres (red) in colon mucosal surfaces layer (green) after 1 hour of rectal administration. Scale bar, 100 µm. The CLSM scanning images of Z axis tomography of wholemount colon tissue were shown in bottom panels. Mucus layer was labeled between dashed lines. (B) Three-dimensional images showing that PS-Cy3 NPs (red) penetrate through the mucus layer (green) and sOKGM-Cy3 (red) microspheres adhere to the mucus layer (green) until 60 mins. Scale bar: 50 µm. (C) Fluorescence images showing localization of PS (labeled by Cy3, red) and miR-31i (labeled by FAM, green) in AOM-DSS induced tumor cells after 12 hours of sOKGM-PS-miR-31i/Cy3 rectal and gavage administration. Scale bar, 100 µm. (D and E) FACS assay showing cellular uptake of PS-Cy3 and PS-TP-Cy3 NPs in HCT116 (D) and LoVo (E) cells with time. The results are reported as the mean ± standard deviation, n = 3, *p < 0.05, **p < 0.01, ***p < 0.001 (F) CLSM images showing intracellular localization of PS-miR-31i/Cy3 and PS-TP-miR-31i/Cy3 NPs in HCT116 cells after 12 hours of incubation with 48 µg/mL (equivalent to PS concentration). PS was labeled by Cy3 (red); miR-31 inhibitor was labeled with FAM (green). Scale bar, 10 µm.

Mucoadhesive properties of the drug delivery system are highly preferable to prolong the retention time of the drugs at the site of absorption, thus improving the therapeutic efficacy and bioavailability 53, 54. Colonic mucosal surfaces highly express cysteine-rich mucins such as MUC2 55, which are critical for mucin-mucin interactions via disulfide bonds 56. In sOKGM-PS delivery system, the cysteine groups on sOKGM microspheres can bind to mucus via disulfide bonds which make the microspheres mucoadhesive. As expected, sOKGM microspheres carried PS-Cy3 NPs were located to colonic mucus layer after 1 hour of rectal administration, while fOKGM-PS-Cy3 microspheres were mostly degraded and released PS-Cy3 NPs which passed through the mucus layer (Figure 4A). Next, upon rectal treatment of sOKGM-Cy3 microspheres or PS-Cy3 NPs, almost all of PS-Cy3 NPs mainly pass through the mucus layer within 60 min, while sOKGM-Cy3 microspheres retained in the mucus layer until at least 60 min (Figure 4B and S4A-B). In contrast, the signals for Cy3-labeled sOKGM (sOKGM-Cy3) microspheres were barely detected in the crypts, while the signals for PS-Cy3 NPs are strongly presented in the crypts (Figure S4C-D). These data suggest that sOKGM-Cy3 microspheres are mucoadhesive, while the encapsulated PSs are mucus-penetrating. Furthermore, we tested the mucoadhesive ability of sOKGM-PS microsphere in the mucus-secreting cell line HT29-MTX. After being incubated with sOKGM-PS-Cy3 for 30 mins, cells still showed strong Cy3 signals merged with mucus after being washed twice with PBS (Figure S4E-F). Taken together, these data demonstrated that the sOKGM-PS microspheres were able to retain in the mucus layer due to its adhesive ability, while the PS-Cy3 NPs can efficiently pass through the mucus layer. The mucus-penetrating feature of PS NPs combined with the mucoadhesive and controlled released feature of sOKGM microspheres, making the sOKGM-PS system suitable for delivering nanomedicine to colonic cells.

### sOKGM-PS-miR-31i/Cur microspheres can efficiently deliver miR-31 inhibitors and Cur to colon tumor cells

In order to evaluate the drug delivery efficiency of sOKGM-PS system in a colitis-associated colon tumor model in mice, we first established the colitis-associated colon tumor model using the AOM-DSS method as shown in Figure S5A. After the colon tumor model was established, mice were administered sOKGM-PS-miR-31i/Cy3 via enema or gavage for 3 consecutive days. The results showed that both FAM-labeled miR-31i and liposoluble Cy3 were targeted to tumor cells after 3 days of administration (Figure 4C). Thus, our findings indicate that sOKGM-PS-miR-31i/Cur microspheres efficiently delivered Cur and the miR-31i to colorectal tumor cells *in vivo* after rectal and oral administration.

The efficient cellular uptake of drugs is another important requirement for their therapeutic application. To enhance the cellular uptake, a peptide (CD133-TP) targeting the colon cancer stem cell surface antigen CD133 was linked to the surface of PSs (PS-TP) 50, 57. Quantification of the internalized fluorescence-activated cell sorting (FACS) analysis showed that the intracellular uptake efficiency of PS-TP NPs was significantly higher than that of PSs in both HCT116 and LoVo cell lines (Figure 4D-E). The internalization efficiency in HCT116 cells (Figure 4D) increased sharply over time and was generally higher than that in LoVo cells (Figure 4E), most likely due to the higher expression level of CD133 in HCT116 cells rather than LoVo cells 58. These data indicate that the peptide targeting CD133 enhanced the uptake of the drug into CRC cells. Next, using CLSM, we further examined the efficiency of PS-TP-miR-31i NPs in delivering the miR-31i and Cur intracellularly to HCT116 and LoVo cells. After 12 hours of incubation with PS-TP-miR-31i/Cy3 NPs, miR-31i (green) and liposoluble Cy3 (red) efficiently entered the cytoplasm of HCT116 and LoVo cells (Figure 4F and Figure S5B), while they only attached to the cell surface of cells treated with PS-miR-31i/Cy3 (without the CD133-TP). In summary, our findings demonstrate that the CD133-TP enhanced the intracellular uptake of PS-TP NPs in colorectal cancer cells.

### miR-31i/Cur-loaded PSs inhibited cancer cell proliferation and tumor growth *in vitro*

**Figure 5:**
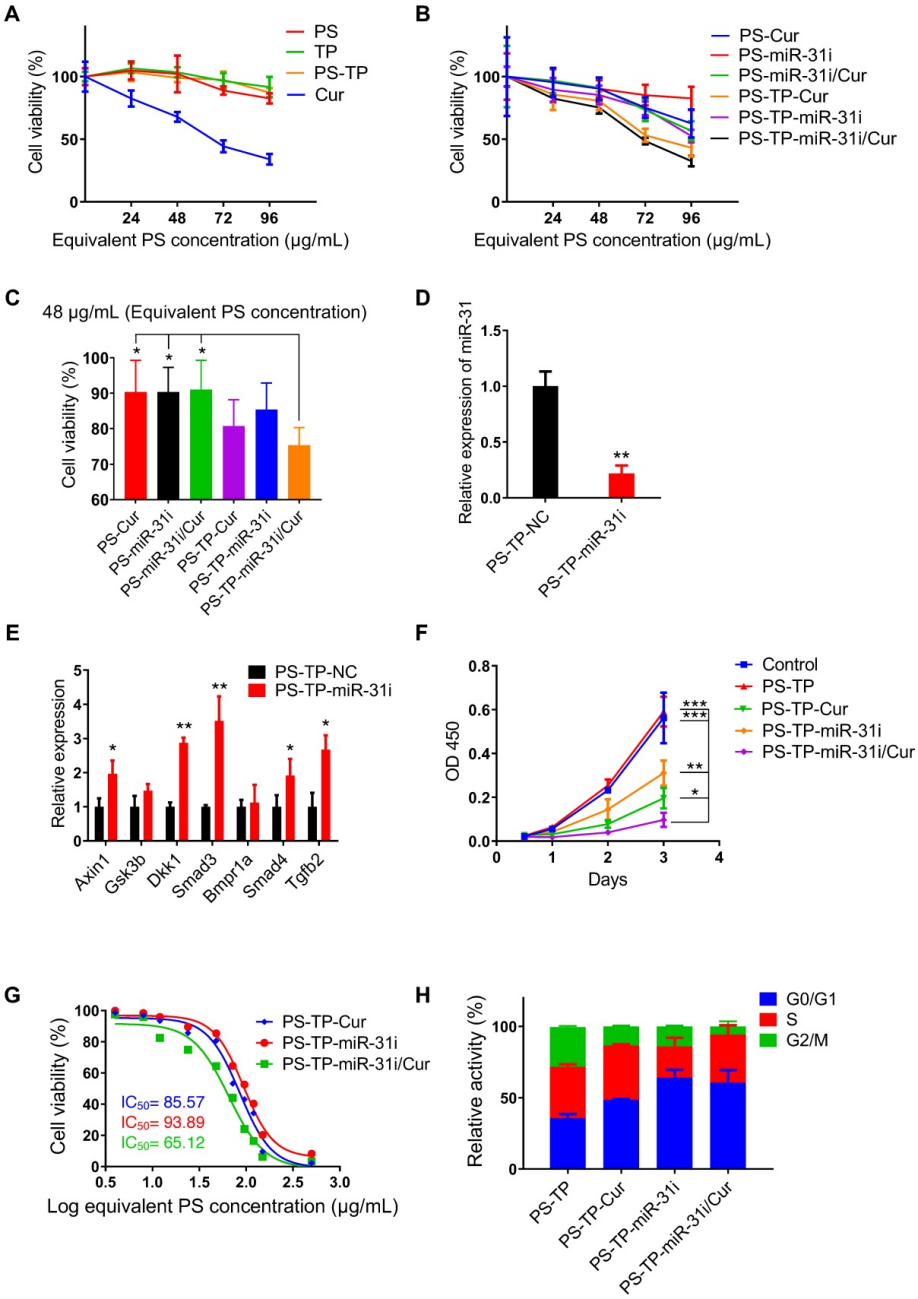
PS-TP-miR-31i/Cur NPs suppressed tumor growth. (A) CCK8 assay showing *in vitro* cytotoxicity profiles of PS, TP, PS-TP, and Cur in HCT116 cells at indicated concentrations after 24 hours of incubation. The results are reported as the mean ± standard deviation, n = 5. (B) CCK8 assay showing *in vitro* cytotoxicity profiles of PS-Cur, PS-miR-31i, PS-miR-31i/Cur, PS-TP-Cur, PS-TP-miR-31i and PS-TP-miR-31i/Cur NPs in HCT116 cells at indicated concentrations after 24 hours of incubation. The results are reported as the mean ± standard deviation, n = 5. (C) CCK8 assay showing *in vitro* cytotoxicity profiles of PS-Cur, PS-miR-31i, PS-miR-31i/Cur, PS-TP-Cur, PS-TP-miR-31i and PS-TP-miR-31i/Cur NPs in HCT116 cells at 48 µg/mL (equivalent to PS concentration) after 24 hours of incubation. The results are reported as the mean ± standard deviation, n = 5, *p < 0.05. (D) qRT-PCR analysis for miR-31 in HCT116 cells after 12 hours of incubation with 48 µg/mL (equivalent to PS concentration) PS-TP-miR-31i NPs. The results are reported as the mean ± standard deviation, n = 3, **p < 0.01. (E) qRT-PCR analysis for miR-31 target genes *Axin1, Gsk3b, Dkk1, Smad3, Bmpr1a, Smad4, Tgfb2* in HCT116 cells after 12 hours of incubation with 48 µg/mL (equivalent to PS concentration) PS-TP-miR-31i NPs. The results are reported as the mean ± standard deviation, n = 3, *p < 0.05, **p < 0.01. (F) CCK-8 assay showing proliferation of HCT116 cells treated with vehicle control, PS-TP-Cur, PS-TP-miR-31i and PS-TP-miR-31i/Cur NPs at the concentration of 48 µg/mL (equivalent to PS concentration) with time. The results are reported as the mean ± standard deviation, n = 5, *p < 0.05, **p < 0.01, ***p < 0.001. (G) The half-maximal inhibitory concentration (IC50) assay of PS-TP-Cur, PS-TP-miR-31i and PS-TP-miR-31i/Cur NPs in HCT116 cells after 24 hours of incubation. The results are reported as the mean ± standard deviation, n = 3. (H) Flow cytometry assay for PI/RNase showing cell cycle pattern of HCT116 cells treated by PS-TP, PS-TP-Cur, PS-TP-miR-31i, PS-TP-miR-31i/Cur NPs for 24 hours. The results are reported as the mean ± standard deviation, n = 3.

The cytotoxicity assay of every component in PS-associated NPs to HCT116 and LoVo cells showed PS, TP and PS-TP NPs had no significant effects on cell viability with increasing concentrations, while Cur decreased cell viability with increasing concentrations (Figure 5A and Figure S6A), indicating that PS-TP NPs had good biocompatibility as a delivery nanocarriers. Next, we examined the effect of miR-31i- and Cur-loaded PSs or PS-TP NPs on cell viability and found that these components exhibited cytotoxicity in a dose-dependent manner (Figure 5B and Figure S6B). Furthermore, the drug loaded PS with CD133 targeting peptide showed higher efficiency on tumor cell toxicity (Figure 5B-C and Figure S6B-C). At concentrations of less than 48 μg/mL (equivalent to the PS concentration), the cell viability of all drug-loaded of PSs formulations were > 75% (Figure 5C and Figure S6C). Thus, we selected 48 μg/mL (equivalent to the PS concentration) as the concentration used to examine the effect on tumor cell proliferation in the following study. As shown in Figure 5D and Figure S6D, the expression of miR-31 was dramatically decreased in PS-TP-miR-31i treated HCT116 and LoVo cells compared to that in PS-TP-NC treated cells.

In addition, the expression levels of the miR-31 target genes Axin1, Gsk3b, Dkk1, Smad3, Bmpr1a, Smad4, and Tgfb2 were upregulated in PS-TP-miR-31i-treated HCT116 and LoVo cells (Figure 5E and Figure S6E). These results indicated that PS-TP-miR-31i NPs successfully deliver miR-31i into CRC cells and effectively regulate the expression of miR-31 target genes. Furthermore, in the tumor cell proliferation detection shown in Figure 5F and Figure S6F, compared with PS-TP-miR-31i and PS-TP-Cur NPs, PS-TP-miR-31i/Cur co-delivery NPs were synergistically suppressed cell growth in combination. Half-maximal inhibitory concentration (IC50) assay indicated that PS-TP-miR-31i/Cur NPs had more remarkable effects on suppressing proliferation of cancer cells than individually loaded miR-31i or Cur (Figure 5G). These findings suggested that the anti-miR-31 oligonucleotides are an effective anti-colon cancer drug, synergistically functioning with Cur. In agreement with these findings, cell cycle analysis showed that treatment with PS-TP-miR-31i and PS-TP-Cur NPs resulted in arrest in the G0/G1 phase (Figure 5H). Specifically, PS-TP-miR-31i had a robust effect on cell cycle arrest, while PS-TP-Cur had only a moderate effect (Figure 5H), indicating that miR-31i and Cur act through distinct mechanisms to suppress cell growth.

### In vivo anti-tumor function of rectal and oral administration of sOKGM-PS-miR-31i/Cur microspheres in the established colon tumor model

**Figure 6:**
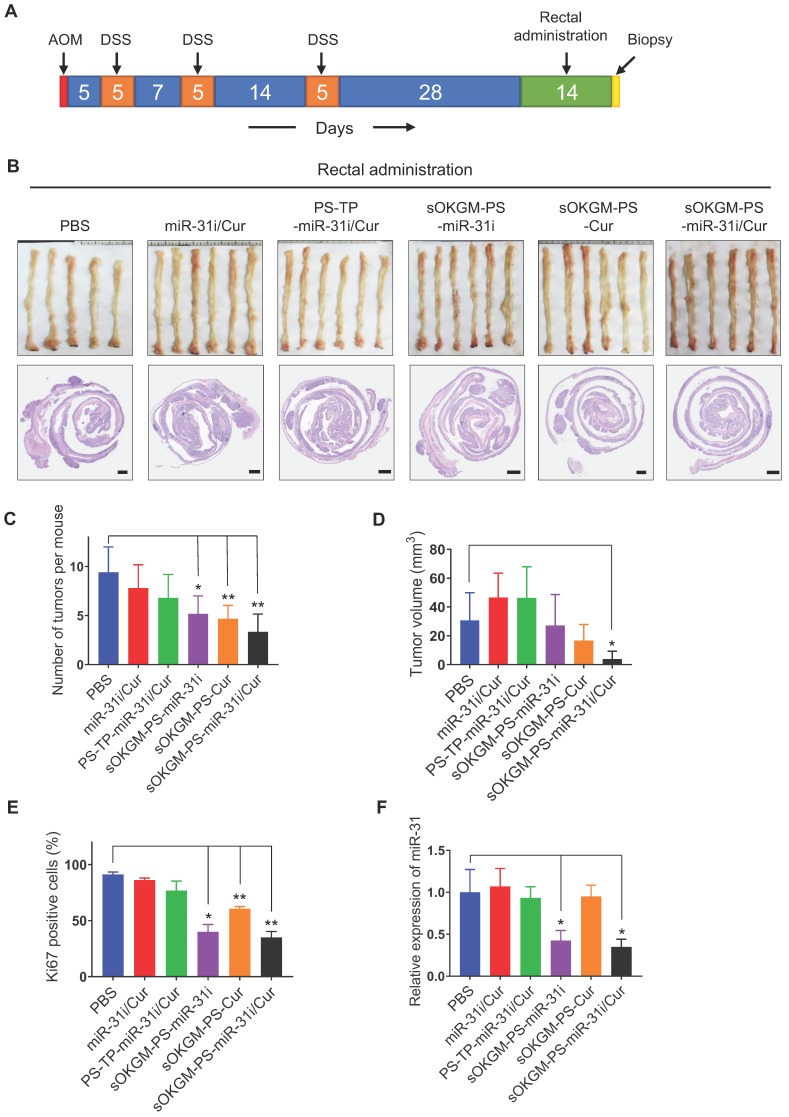
Rectal delivery of sOKGM-PS-miR-31i/Cur microspheres suppressed colon tumor growth *in vivo*. (A) Schematics for rectal delivery of sOKGM-PS-miR-31i/Cur microspheres in treating AOM-DSS induced colon tumors. (B) Gross (upper panels) and Hematoxylin & eosin staining (bottom panels) images of colon tumors from mice rectally treated with indicated formulations for 2 weeks. Scale bar, 1 mm. (C and D) Quantification of tumor numbers (C) and volume (D) shown in panel B. The results are reported as the mean ± standard deviation (PBS, n = 5; n = 6 for the other treatments), *p < 0.05; **p < 0.01. (E) Quantification of the percentage of Ki67^+^ cells in tumors as shown in Figure S7A. The results are reported as the mean ± standard deviation (PBS, n = 5; n = 6 for the other treatments), *p < 0.05; **p < 0.01. (F) qRT-PCR analysis for miR-31 in colon tumors from mice rectally treated with indicated formulations for 2 weeks. The results are reported as the mean ± standard deviation (PBS, n = 5; n = 6 for the other treatments), *p < 0.05.

Before our system can be used as an effective therapeutic delivery approach for CRC treatment, the *in vivo* effects of the rectal and oral administration of sOKGM-PS-miR-31i/Cur microspheres in established tumor models are important to evaluate. To this end, we established the AOM-DSS tumor model of inflammation-driven colorectal adenocarcinoma, which expressing CD133 and much closer to human colon cancer compared with Xenografted tumor 59. Twenty-eight days after the third round of DSS treatment, we treated the mice once daily for 14 consecutive days with various formulations via rectal administration (Figure 6A). Like PBS treatment, the treatments of miR-31i/Cur and PS-PT-miR-31i/Cur without mucoadhesive microspheres did not affect tumor growth (Figure 6B-D). However, treatment with drug-loaded PS-TP-NPs carried with sOKGM microspheres resulted in a reduction in tumor numbers and volumes, although the volumes reduction were nonsignificant in treatment of sOKGM-PS-miR-31i and sOKGM-PS-Cur microspheres (Figure 6C-D). Consistent with this result, treatment with these microspheres markedly suppressed cell proliferation (Figure 6E and Figure S7A). Strikingly, sOKGM-PS-miR-31i/Cur microspheres treatment significantly reduced the tumor numbers and the average volume of each tumor (Figure 6B-D), concomitant with a decrease in proliferative cells (Figure 6E and Figure S7A). In contrast, PS-TP-miR-31i/Cur NPs cannot significantly suppress tumor growth, most likely due to its short retention time in the colon. The suppression of miR-31 upon the treatments of sOKGM-PS-miR-31i and sOKGM-PS-miR-31i/Cur was confirmed (Figure 6F), indicating that anti-miR-31 oligonucleotides work well in colonic tumor cells. Together, these data indicate that sOKGM-PS-miR-31i/Cur microspheres are the most effective formulation for treating CRC via rectal administration.

**Figure 7:**
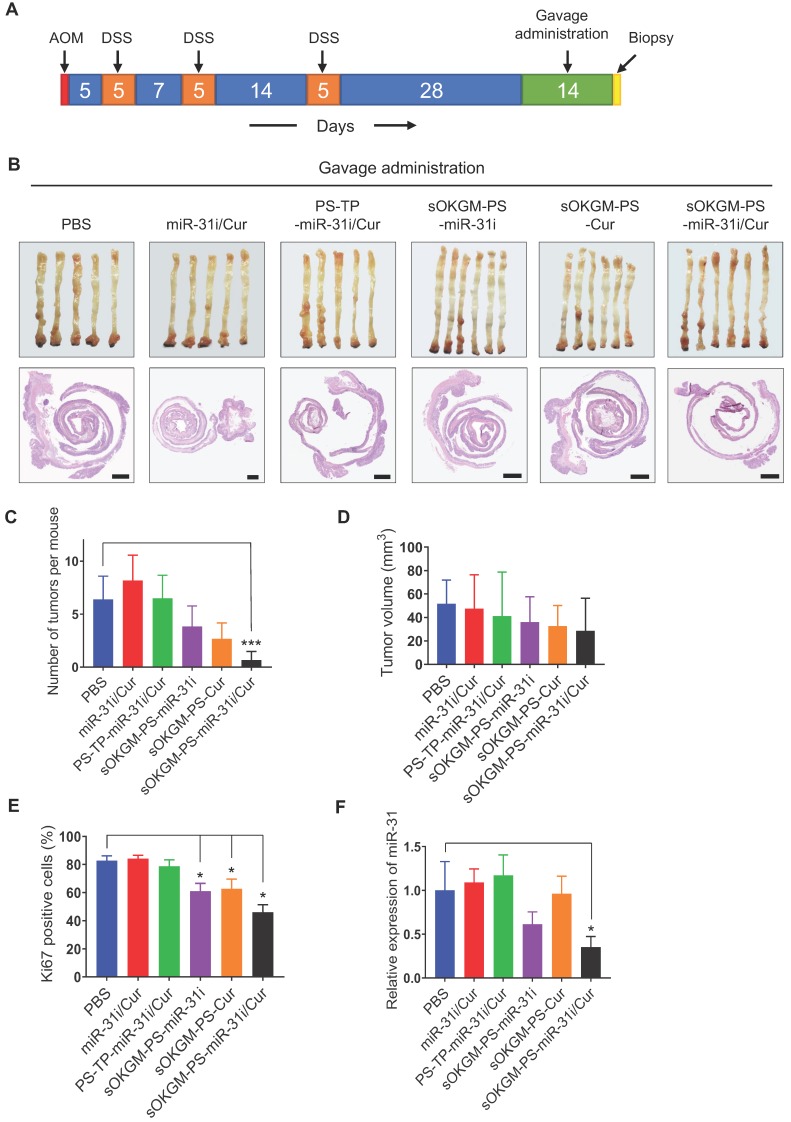
Gavage delivery of sOKGM-PS-miR-31i/Cur microspheres suppressed colon tumor growth *in vivo*. (A) Schematics for gavage delivery of sOKGM-PS-miR-31i/Cur microspheres in treating AOM-DSS induced colon tumors. (B) Gross (upper) and Hematoxylin & eosin staining (bottom) images of colon tumors from mice gavage-treated with indicated formulations for 2 weeks. Scale bar, 1 mm. (C and D) Quantification of tumor numbers (C) and volume (D) shown in panel B. The results are reported as the mean ± standard deviation (PBS, n = 5; n = 6 for the other treatments), ***p < 0.001. (E) Quantification of the percentage of Ki67^+^ cells in tumors as shown in Figure S7B. The results are reported as the mean ± standard deviation (PBS, n = 5; n = 6 for the other treatments), *p < 0.05. (F) qRT-PCR analysis for miR-31 in colon tumors from mice gavage-treated with indicated formulations for 2 weeks. The results are reported as the mean ± standard deviation (PBS, n = 5; n = 6 for the other treatments), *p < 0.05.

Furthermore, considering that sOKGM-PS-miR-31i/Cur microspheres can pass through the GI tract and reach the colon (Figure 3C and Figure S3A); we investigated whether these microspheres are also effective as an orally administered delivery system. After two weeks of gavage (Figure 7A), sOKGM-PS-miR-31i/Cur microspheres exhibited the best anti-tumor effect compared to those of the other treatments, as indicated by the decrease in tumor numbers and volumes, although the decrease in the tumor volumes was not statistically significant (Figure 7B-E and Figure S7B). And the miR-31 also significantly suppressed by oral treatment of sOKGM-PS-miR-31i and sOKGM-PS-miR-31i/Cur microspheres (Figure 7F). These findings suggest that sOKGM-PS-miR-31i/Cur microspheres can also be an orally administered delivery system for treating CRC. We noticed that the inhibitory efficacy of oral administration was less effective than that of rectal administration. We believe that sOKGM-PS-miR-31i/Cur microspheres may be partially retained at the small intestinal mucus, and degraded by digestive solution in the small intestine, thus reducing the drug amounts arriving to colon sites. Therefore, to develop the microsphere as a colon-specific oral delivery system, the microspheres need to be further optimized.

## Conclusions

In summary, we developed sOKGM-PS-miR-31i/Cur microspheres as an ideal rectal and oral delivery system for nanomedicines with advantages of mucoadhesive and mucus-penetrating properties. In this system, double cross-linked sOKGM microspheres were more stable and mucoadhesive, and thus exhibited prolonged retention at colorectal sites, resulting in improved drug bioavailability. The released PS-miR-31i/Cur can efficiently co-deliver miR-31i and Cur to colon tumors, and thus suppressing colon tumor growth *in vivo*. Based on the low cost and toxicity of basic materials (α-lactalbumin and konjac glucomannan are all food resources), straightforward making process, as well as the stable property for long time storage, our delivery system is viability of industrial fabrication.

## Supplementary Material

Supplementary figures and tables.
