# Supplementary material for: Mucoadhesive-to-penetrating controllable peptosomes-in-microspheres co-loaded with anti-miR-31 oligonucleotide and Curcumin for targeted colorectal cancer therapy
Source: Theranostics. 2020 Feb 18;10(8):3594–611. doi: 10.7150/thno.40318 (PMC7069075; doi:10.7150/thno.40318)
Supplement: Supplementary file 1 — Supplementary figures and tables. [file thnov10p3594s1.pdf]

Supporting Information

Mucoadhesive-to-penetrating controllable peptosomes-in-microspheres co-loaded with anti-miR-31 oligonucleotide and Curcumin for targeted colorectal cancer therapy

Ran Zhao[1, 2, #], Sujuan Du[1, #], Ying Liu[3], Cong Lv[1, 2], Yongli Song[1, 2], Xinchun Chen[2], Bing Zhang[4], Dan Li[3], Shan Gao[5], Wei Cui[6], Maksim V. Plikus[7], Xiaohua Hou[8], Kaichun Wu[9], Zhanju Liu[10], Zhihua Liu[11], Yingzi Cong[12], Yuan Li[3, *], and Zhengquan Yu[1, *]

1. State Key Laboratories for Agrobiotechnology and Beijing Advanced Innovation Center for Food Nutrition and Human Health, College of Biological Sciences, China Agricultural University, Beijing, 100193, China.
2. Guangdong Provincial Key Laboratory of Regional Immunity and School of Medicine, Shenzhen University, Shenzhen, 518055, China.
3. Beijing Advanced Innovation Center for Food Nutrition and Human Health, Key Laboratory of Functional Dairy, College of Food Science and Nutritional Engineering, China Agricultural University, Beijing, 100083, China.
4. College of Veterinary Medicine, China Agricultural University, Beijing, 100193, China.
5. CAS Key Laboratory of Bio-medical Diagnostics, Suzhou Institute of Biomedical Engineering and Technology, Chinese Academy of Sciences, Suzhou, Jiangsu, 215163, China.

6. Institute of Reproductive and Developmental Biology, Faculty of Medicine, Imperial College London, W12 0NN, UK.
7. Department of Developmental and Cell Biology, Sue and Bill Gross Stem Cell Research center, Center for Complex Biological Systems, University of California, Irvine, Irvine, CA 92697, USA.
8. Division of Gastroenterology, Union Hospital, Tongji Medical College, Huazhong University of Technology and Science, Wuhan, 430022, China.
9. Department of Gastroenterology, Xijing Hospital, The Fourth Military Medical University, Xi'an, 710032, China.
10. Department of Gastroenterology, The Shanghai Tenth People's Hospital, Tongji University, Shanghai, 200072, China.
11. State Key Laboratory of Molecular Oncology, National Cancer Center/Cancer Hospital, Chinese Academy of Medical Sciences, Beijing, 100021, China.
12. Department of Microbiology and Immunology, University of Texas Medical Branch, Galveston, TX 78701, USA.

#These authors contributed equally.

*Co-corresponding author: Zhengquan Yu and Yuan Li.

State Key Laboratories for Agrobiotechnology and Beijing Advanced Innovation Center for Food Nutrition and Human Health, College of Biological Sciences, China Agricultural University, Yuanming West Rd. 2, Haidian District, Beijing, 100193, China.

Tel: 86-10-62734420; FAX: 86-10-62733904;

E-mail: zyu@cau.edu.cn; yuanli@cau.edu.cn

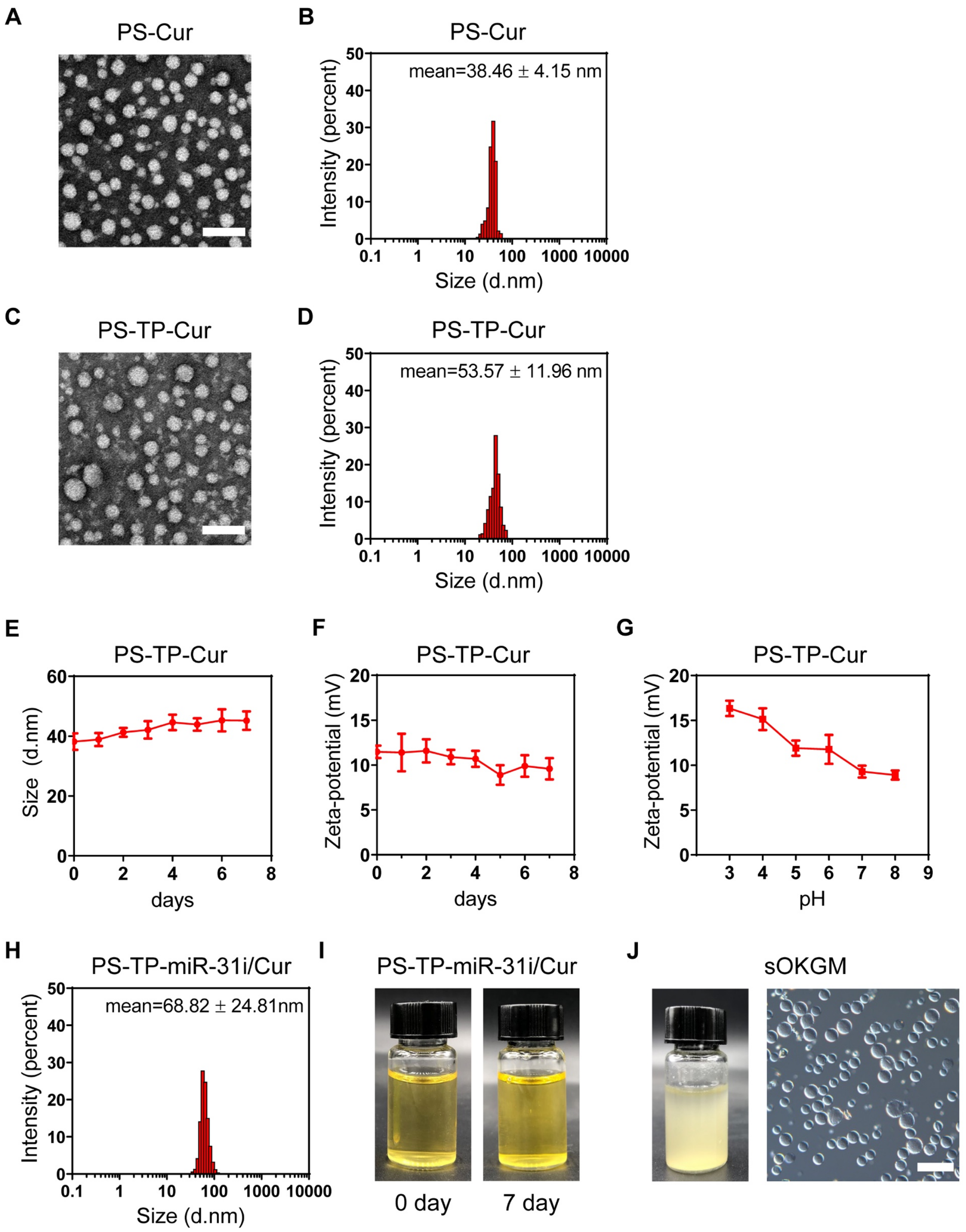
A
PS-Cur
B
PS-Cur
mean=38.46 ± 4.15 nm
Intensity (percent)
Size (d.nm)
C
PS-TP-Cur
D
PS-TP-Cur
mean=53.57 ± 11.96 nm
Intensity (percent)
Size (d.nm)
E
PS-TP-Cur
Size (d.nm)
days
F
PS-TP-Cur
Zeta-potential (mV)
days
G
PS-TP-Cur
Zeta-potential (mV)
pH
H
PS-TP-miR-31i/Cur
mean=68.82 ± 24.81nm
Intensity (percent)
Size (d.nm)
I
PS-TP-miR-31i/Cur
0 day
7 day
J
sOKGM

Figure S1. (A) TEM images of PS-Cur NPs. Scale bars, 100 nm. (B) The size distributions of PS-Cur NPs in diameter. (C) TEM images of PS-TP-Cur NPs. Scale bars, 100 nm. (D) The size distributions of PS-TP-Cur NPs in diameter. (E) The dynamic change of the size of PS-TP-Cur NPs with time. n = 3. (F) The dynamic change of the zeta potential of PS-TP-Cur NPs with time. n = 3. (G) The dynamic change of the zeta potential of PS-TP-Cur NPs with pH values. n = 3. The results are reported as the mean ± standard deviation. (H) The size distributions of PS-TP-miR-31i/Cur NPs in diameter. (I) Gross images of PS-TP-miR-31i/Cur NPs aqueous solution after 0 and 7 days of storage at 37°C. (J) Gross image of sOKGM microspheres aqueous solution (left), and microscopy photo (right). Scale bars, 50 μm.

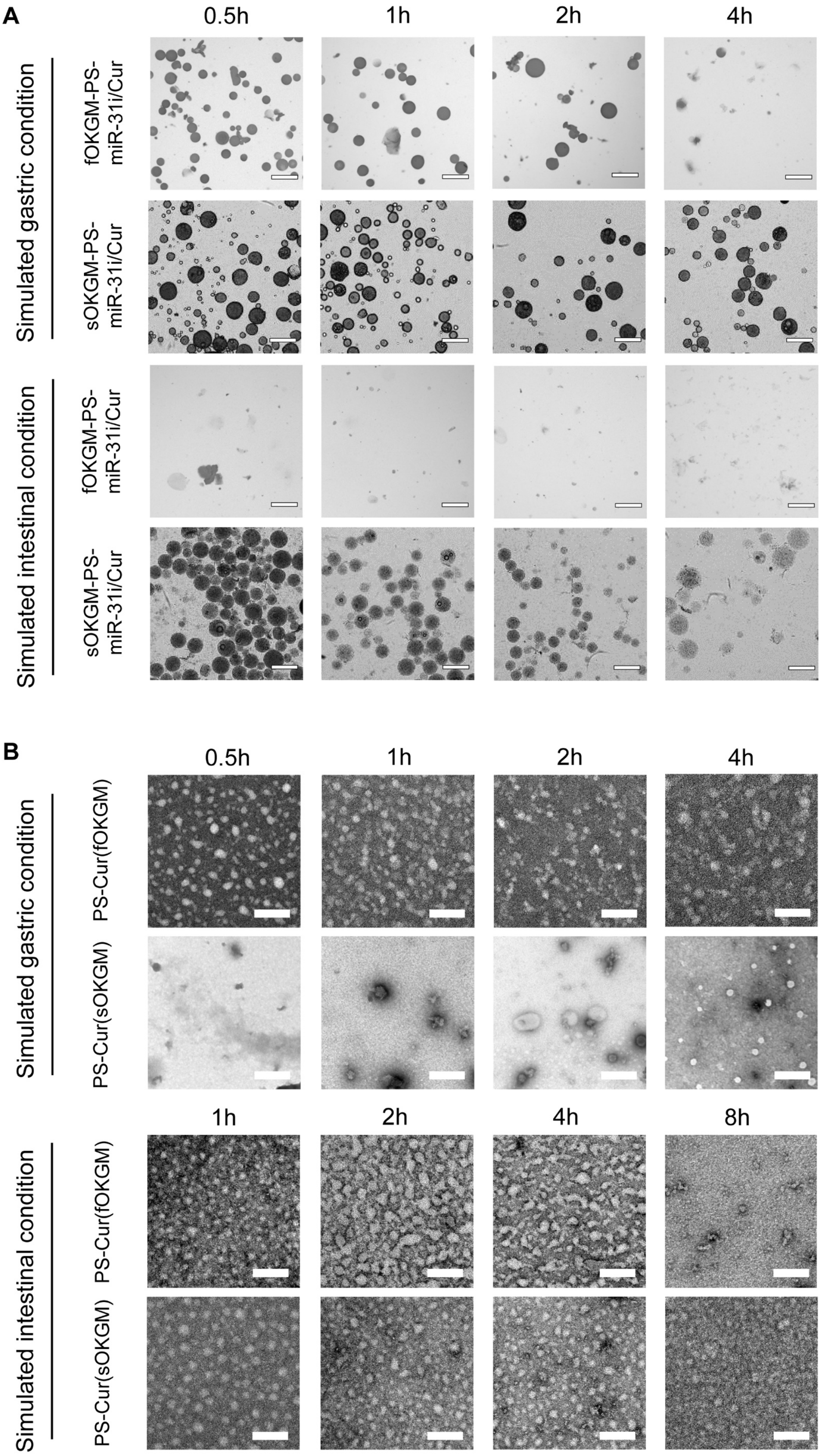
A
0.5h
1h
2h
4h
Simulated gastric condition
fOKGM-PS-miR-31i/Cur
sOKGM-PS-miR-31i/Cur
Simulated intestinal condition
fOKGM-PS-miR-31i/Cur
sOKGM-PS-miR-31i/Cur
B
0.5h
1h
2h
4h
Simulated gastric condition
PS-Cur(fOKGM)
PS-Cur(sOKGM)
1h
2h
4h
8h
Simulated intestinal condition
PS-Cur(fOKGM)
PS-Cur(sOKGM)

Figure S2. (A) The dynamic change of sOKGM-PS-miR-31i/Cur and fOKGM-PS-miR-31i/Cur microspheres in simulated gastric and intestinal conditions with time. Scale bar, 50 μm. (B) The TEM images of the collected supernatant showed that the PSs are released from sOKGM-PS-miR-31i/Cur and fOKGM-PS-miR-31i/Cur microspheres in simulated gastric and intestinal conditions over time. Scale bar, 200 nm.

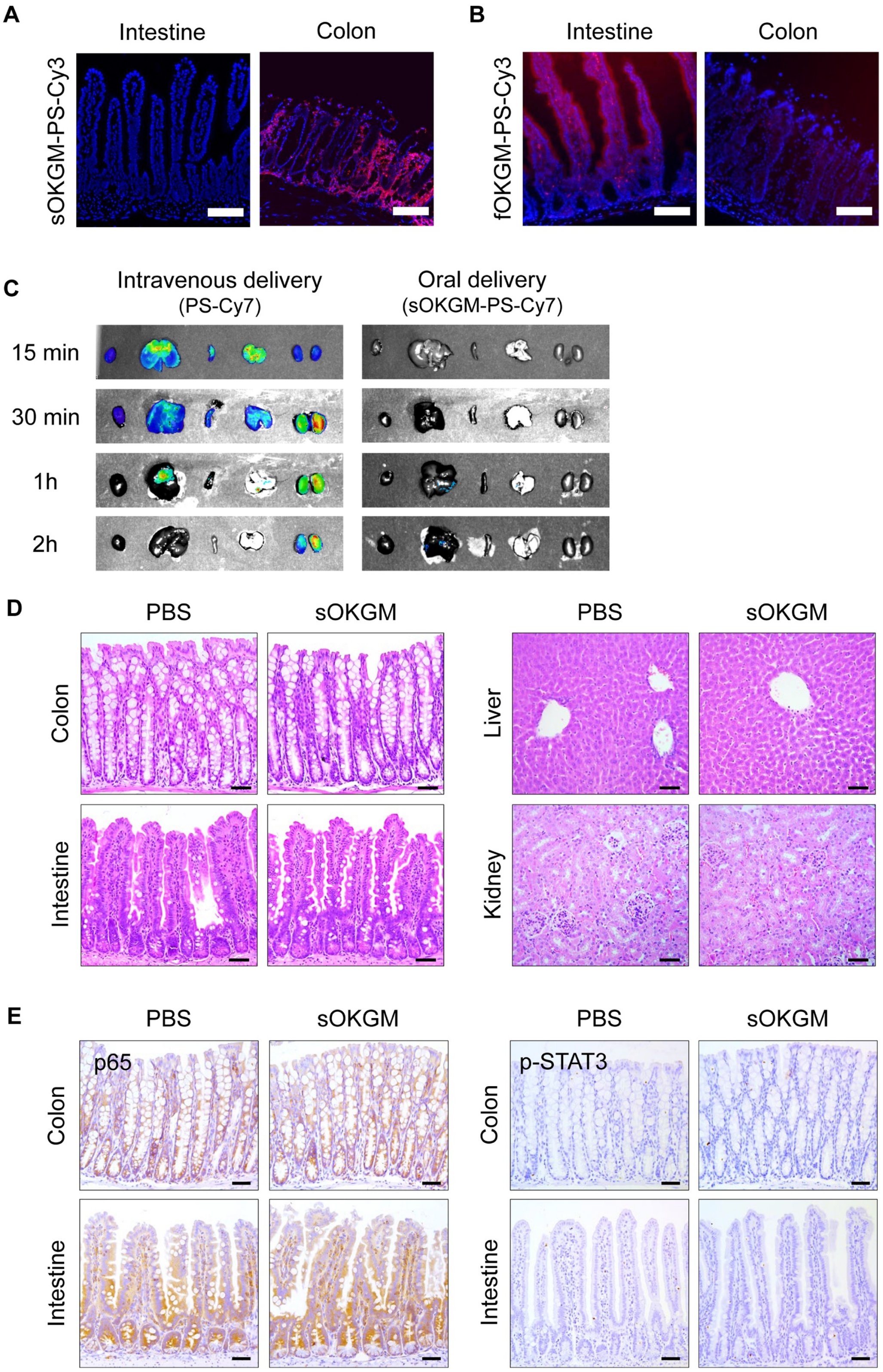
A
Intestine
Colon
sOKGM-PS-Cy3
B
Intestine
Colon
fOKGM-PS-Cy3
C
Intravenous delivery
(PS-Cy7)
Oral delivery
(sOKGM-PS-Cy7)
15 min
30 min
1h
2h
D
PBS
sOKGM
PBS
sOKGM
Colon
Intestine
Liver
Kidney
E
PBS
sOKGM
PBS
sOKGM
p65
p-STAT3
Colon
Intestine
Colon
Intestine

Figure S3. (A and B) The distribution of sOKGM-PS-Cy3 (A) and fOKGM-PS-Cy3 (B) microspheres in intestinal and colon tissues after 12 hours of gavage administration. PS labeled by Cy-3 (red). Scale bar, 200 μm. (C) Distribution of PS-Cy7 NPs in heart, liver, spleen, lung and kidney after intravenous administration of PS-Cy7 (left) and oral administration of sOKGM-PS-Cy7 (right) over time, PS labeled by Cy-7. (D) Hematoxylin & eosin staining of mouse colon, intestine liver and kidney after oral treatment with PBS or sOKGM-PS microspheres once daily for 7 consecutive days. Scale bars, 100 μm. (E) Immunohistochemistry for p65 and p-STAT in mouse colon and intestine after oral treatment with PBS or sOKGM-PS microspheres once daily for 7 consecutive days. Scale bars, 100 μm.

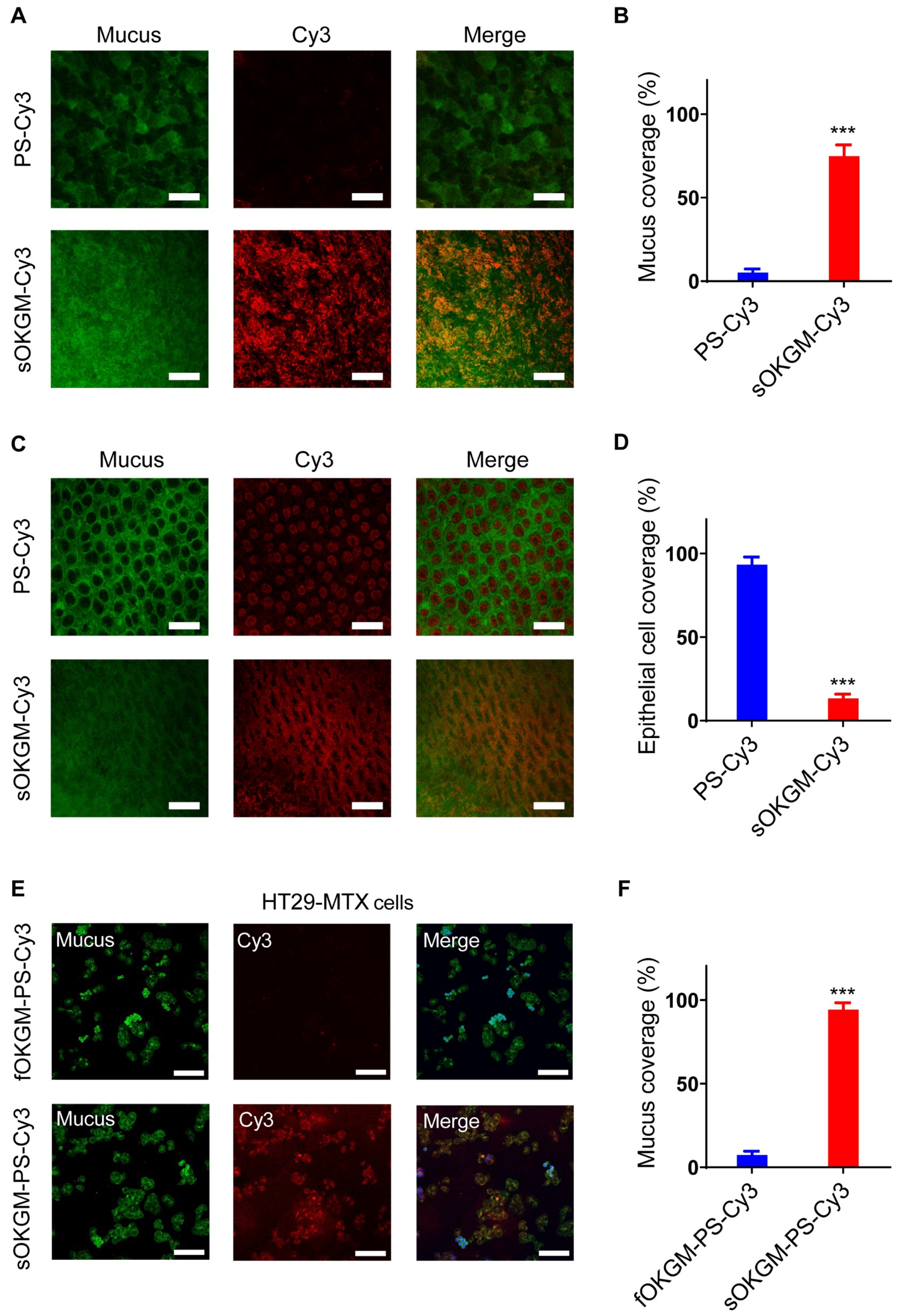
A
Mucus
Cy3
Merge
PS-Cy3
sOKGM-Cy3
B
Mucus coverage (%)
100
50
0
***
PS-Cy3
sOKGM-Cy3
C
Mucus
Cy3
Merge
PS-Cy3
sOKGM-Cy3
D
Epithelial cell coverage (%)
100
50
0
***
PS-Cy3
sOKGM-Cy3
E
HT29-MTX cells
fOKGM-PS-Cy3
sOKGM-PS-Cy3
Mucus
Cy3
Merge
F
Mucus coverage (%)
100
50
0
***
fOKGM-PS-Cy3
sOKGM-PS-Cy3

Figure S4. (A) CLSM images of PS-Cy3 and sOKGM-Cy3 microspheres (red) in colon mucus layer (green) after 1 hour of rectal administration. Scale bar, 100 μm. (B) Quantification of the percentage of Cy3 signal coverage in colon mucus layer in panel A. The results are reported as the mean ± standard deviation, n = 3, ***p < 0.001. (C) CLSM images of PS-Cy3 NPs and sOKGM-Cy3 microspheres (red) in colon crypts located under mucus layer after 1 hour of rectal administration. Scale bar, 100 μm. (D) Quantification of the Cy3 signal coverage in the colon epithelial cells in panel C. The results are reported as the mean ± standard deviation, n = 3, ***p < 0.001. (E) CLSM images of fOKGM-PS-Cy3 or sOKGM-PS-Cy3 (red) microsphere, and immunofluorescence for WGA (green) in HT29-MTX cells after 30 min of incubation with fOKGM-PS-Cy3 or sOKGM-PS-Cy3 microspheres. Scale bar, 100 μm. (F) Quantification of the Cy3 signal coverage in the mucus surface of HT29-MTX cells in panel A. The results are reported as the mean ± standard deviation, n = 3, ***p < 0.001.

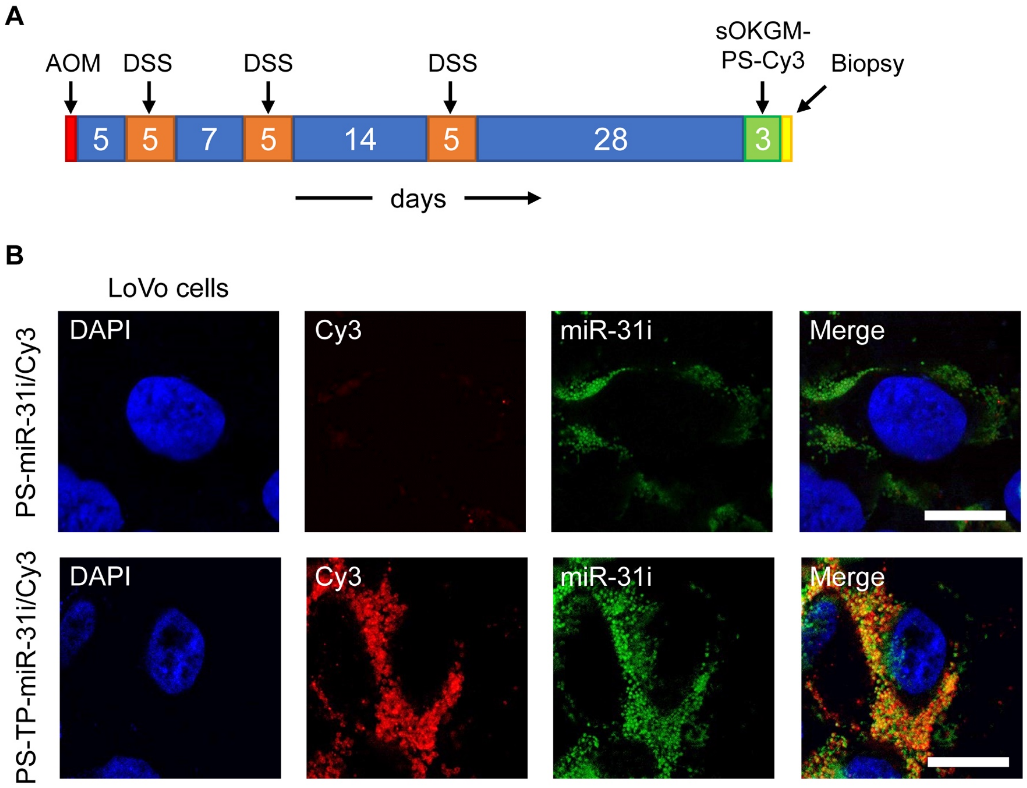
A
AOM
DSS
DSS
DSS
sOKGM-
PS-Cy3
Biopsy
5
5
7
5
14
5
28
3
days
B
LoVo cells
PS-miR-31i/Cy3
PS-TP-miR-31i/Cy3
DAPI
Cy3
miR-31i
Merge

Figure S5. (A) Schematics for rectal delivery of sOKGM-PS-Cy3 microspheres in treating AOM-DSS induced colon tumors for 3 days. (B) CLSM images showing intracellular localization of PS-miR-31i/Cy3 and PS-TP-miR-31i/Cy3 NPs in LoVo cells after 12 hours of incubation. PSs were labeled by Cy3 (red); miR-31i was labeled with FAM (green). Scale bar, 10 μm.

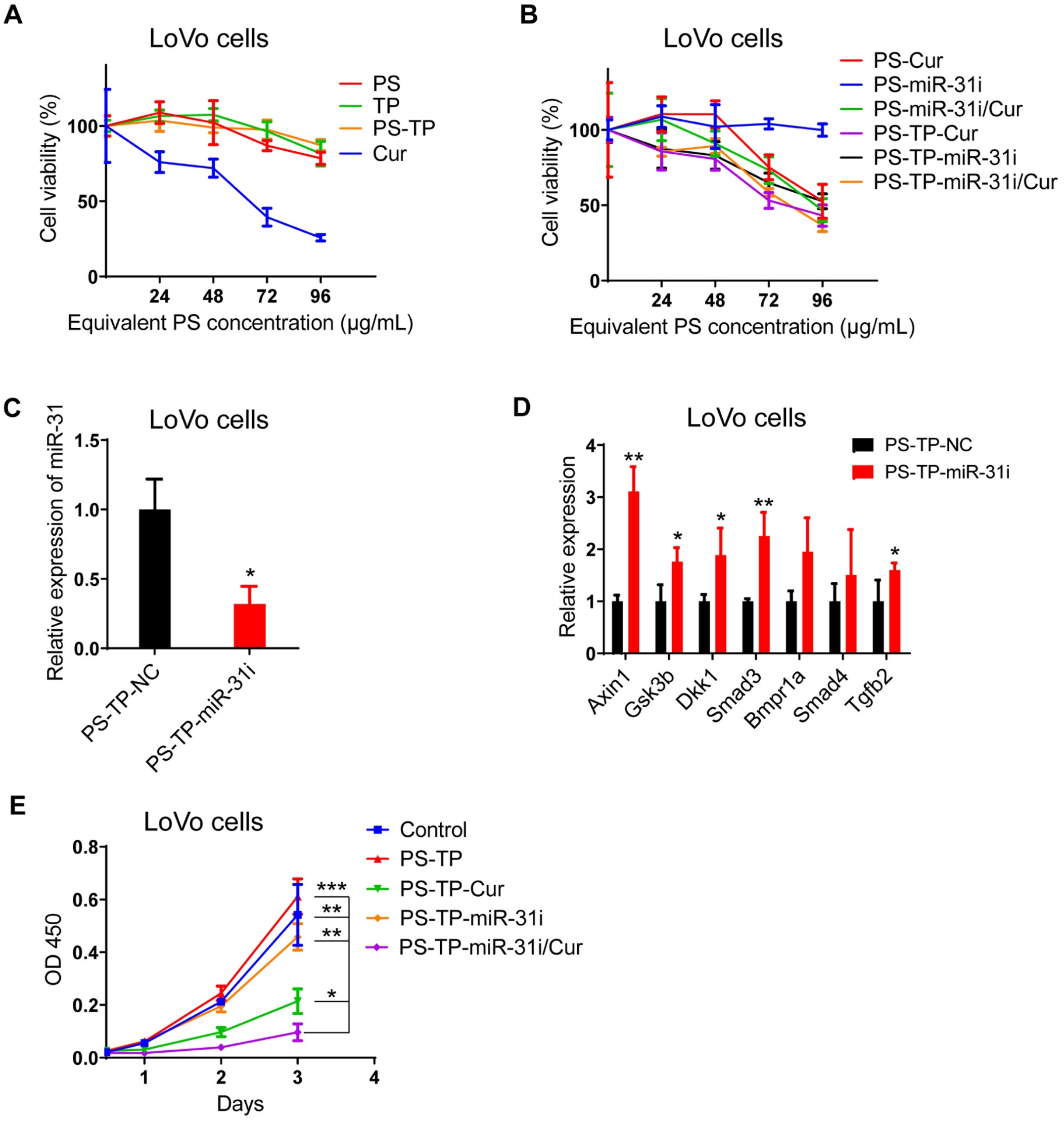
A
LoVo cells
Cell viability (%)
Equivalent PS concentration (μg/mL)
PS
TP
PS-TP
Cur
B
LoVo cells
Cell viability (%)
Equivalent PS concentration (μg/mL)
PS-Cur
PS-miR-31i
PS-miR-31i/Cur
PS-TP-Cur
PS-TP-miR-31i
PS-TP-miR-31i/Cur
C
LoVo cells
Relative expression of miR-31
PS-TP-NC
PS-TP-miR-31i
D
LoVo cells
Relative expression
PS-TP-NC
PS-TP-miR-31i
Axin1
Gsk3b
Dkk1
Smad3
Bmpr1a
Smad4
Tgfb2
E
LoVo cells
OD 450
Days
Control
PS-TP
PS-TP-Cur
PS-TP-miR-31i
PS-TP-miR-31i/Cur

Figure S6. (A) CCK8 assay showing in vitro cytotoxicity profiles of PS, TP, PS-TP, and Cur in LoVo cells at indicated concentrations after 24 hours of incubation. The results are reported as the mean ± standard deviation, n = 3. (B) CCK8 assay showing in vitro cytotoxicity profiles of PS-Cur, PS-miR-31i, PS-miR-31i/Cur, PS-TP-Cur, PS-TP-miR-31i and PS-TP-miR-31i/Cur NPs in LoVo cells at indicated concentrations after 24 hours of incubation. The results are reported as the mean ± standard deviation, n = 3. (C) CCK8 assay showing in vitro cytotoxicity profiles of PS-Cur, PS-miR-31i, PS-miR-31i/Cur, PS-TP-Cur, PS-TP-miR-31i and PS-TP-miR-31i/Cur NPs in LoVo cells at 48 μg/mL (equivalent to PS concentration) after 24 hours of incubation. The results are reported as the mean ± standard deviation, n = 3, *$p < 0.05$. (D) qRT-PCR analysis for miR-31 in LoVo cells after 12 hours of incubation with 48 μg/mL (equivalent to PS concentration) PS-TP-miR-31i NPs. The results are reported as the mean ± standard deviation, n = 3, *$p < 0.05$. (E) qRT-PCR analysis for miR-31 target genes *Axin1, Gsk3b, Dkk1, Smad3, Bmpr1a, Smad4, Tgfb2* in LoVo cells after 12 hours of incubation with 48 μg/mL (equivalent to PS concentration) PS-TP-miR-31i NPs. The results are reported as the mean ± standard deviation, n = 3, *$p < 0.05$, **$p < 0.01$. (F) CCK-8 assay showing proliferation of LoVo cells treated with vehicle control, PS-TP-Cur, PS-TP-miR-31i and PS-TP-miR-31i/Cur NPs at the concentration of 48 μg/mL (equivalent to PS concentration) with time. The results are reported as the mean ± standard deviation, n = 5, *$p < 0.05$; **$p < 0.01$; ***$p < 0.01$.

A

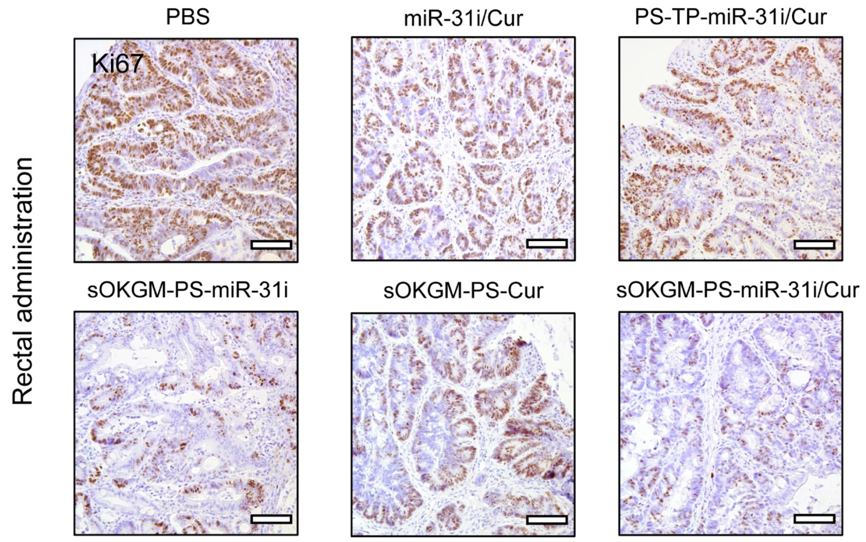
PBS
miR-31i/Cur
PS-TP-miR-31i/Cur
Ki67
Rectal administration
sOKGM-PS-miR-31i
sOKGM-PS-Cur
sOKGM-PS-miR-31i/Cur

B

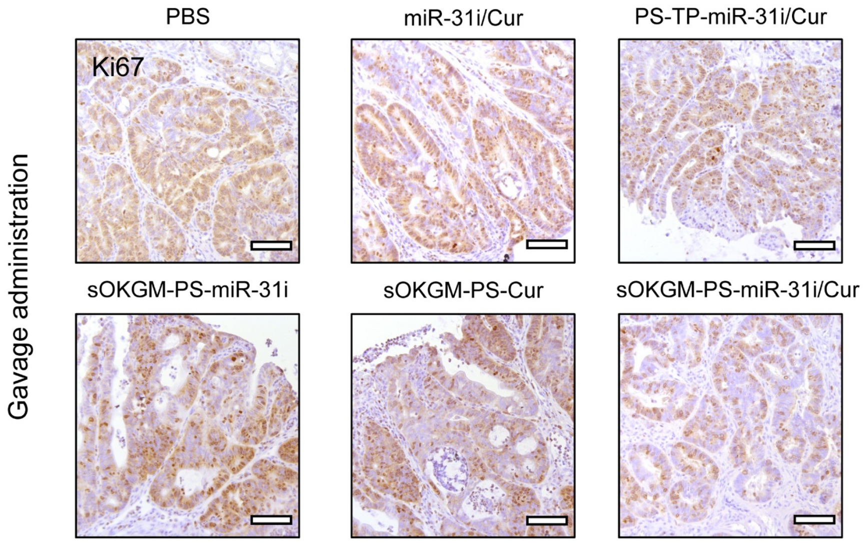
PBS
miR-31i/Cur
PS-TP-miR-31i/Cur
Ki67
Gavage administration
sOKGM-PS-miR-31i
sOKGM-PS-Cur
sOKGM-PS-miR-31i/Cur

Figure S7. (A) Immunohistochemistry for ki67 in AOM-DSS induced colon tumors from mice after 2 weeks of rectal treatment with indicated formulations. Scale bar, 200 μm. (B) Immunohistochemistry for ki67 in AOM-DSS induced colon tumors from mice after 2 weeks of gavage treatment with indicated formulations. Scale bar, 200 μm.

Table S1. The zeta potential of PS complexes.

	Zeta-potential (mV)
PS-Cur	-14.82 ± 1.53
PS-TP-Cur	11.49 ± 0.73
PS-TP-miR-31/Cur	1.33 ± 0.86

The results are reported as the mean ± standard deviation, n = 3.
